# Synergistic cross‐talk of hedgehog and interleukin‐6 signaling drives growth of basal cell carcinoma

**DOI:** 10.1002/ijc.31724

**Published:** 2018-10-01

**Authors:** Christina Sternberg, Wolfgang Gruber, Markus Eberl, Suzana Tesanovic, Manuela Stadler, Dominik P. Elmer, Michaela Schlederer, Sandra Grund, Simone Roos, Florian Wolff, Supreet Kaur, Doris Mangelberger, Hans Lehrach, Hendrik Hache, Christoph Wierling, Josef Laimer, Peter Lackner, Markus Wiederstein, Maria Kasper, Angela Risch, Peter Petzelbauer, Richard Moriggl, Lukas Kenner, Fritz Aberger

**Affiliations:** ^1^ Department of Biosciences, Cancer Cluster Salzburg Paris‐Lodron University of Salzburg Salzburg Austria; ^2^ Clinical Institute of Pathology Medical University of Vienna Vienna Austria; ^3^ Unit Laboratory Animal Pathology University of Veterinary Medicine Vienna Vienna Austria; ^4^ CytoSwitch Munich Germany; ^5^ Department of Vertebrate Genomics Max Planck Institute for Molecular Genetics Berlin Germany; ^6^ Alacris Theranostics GmbH Berlin Germany; ^7^ Department of Biosciences and Nutrition and Center for Innovative Medicine Karolinska Institutet Huddinge Sweden; ^8^ Department of Dermatology Medical University of Vienna Vienna Austria; ^9^ Ludwig Boltzmann Institute for Cancer Research Vienna Austria; ^10^ Institute of Animal Breeding and Genetics University of Veterinary Medicine Vienna Vienna Austria; ^11^ Medical University Vienna Vienna Austria

**Keywords:** basal cell carcinoma, GLI transcription factors, hedgehog, GLI signaling, interleukin‐6 signaling, STAT transcription factors

## Abstract

Persistent activation of hedgehog (HH)/GLI signaling accounts for the development of basal cell carcinoma (BCC), a very frequent nonmelanoma skin cancer with rising incidence. Targeting HH/GLI signaling by approved pathway inhibitors can provide significant therapeutic benefit to BCC patients. However, limited response rates, development of drug resistance, and severe side effects of HH pathway inhibitors call for improved treatment strategies such as rational combination therapies simultaneously inhibiting HH/GLI and cooperative signals promoting the oncogenic activity of HH/GLI. In this study, we identified the interleukin‐6 (IL6) pathway as a novel synergistic signal promoting oncogenic HH/GLI via STAT3 activation. Mechanistically, we provide evidence that signal integration of IL6 and HH/GLI occurs at the level of *cis‐*regulatory sequences by co‐binding of GLI and STAT3 to common HH‐IL6 target gene promoters. Genetic inactivation of Il6 signaling in a mouse model of BCC significantly reduced *in vivo* tumor growth by interfering with HH/GLI‐driven BCC proliferation. Our genetic and pharmacologic data suggest that combinatorial HH‐IL6 pathway blockade is a promising approach to efficiently arrest cancer growth in BCC patients.

## Abstract 2

**What's new?**

Persistent activation of hedgehog (HH)/GLI signaling represents the main driver signal for the development of basal cell carcinoma (BCC), a common non‐melanoma skin cancer with rising incidence. Small molecule hedgehog pathway inhibitors are successfully used for the treatment of hedgehog‐driven BCC, but frequent drug resistance calls for improved strategies. Here, the authors identified the interleukin‐6 pathway as a novel synergistic signal promoting oncogenic HH/GLI via STAT3 activation. The synergistic interaction was required for the *in vivo* growth of hedgehog‐driven BCC. The study thus provides a rationale for effective combination treatments simultaneously targeting oncogenic hedgehog and interleukin‐6 signaling in BCC patients.

**Abbreviations**
BCC: basal cell carcinoma
ChIP: chromatin immunoprecipitation
EDN2: endothelin 2
EGFR: epidermal growth factor receptor
ERK: extracellular signal‐regulated kinase
GLI: glioma‐associated oncogene
GSEA: gene set enrichment analysis
HH: hedgehog
IL6: interleukin‐6
IL6R: interleukin‐6 receptor
JAK: Janus tyrosine kinase
MEK: mitogen‐activated protein/extracellular signal‐regulated kinase kinase
NFkB: nuclear factor of kappa light polypeptide gene enhancer in B‐cells
NRP1: neuropilin 1
OSM: oncostatin M
PI3K: phosphatidylinositol 3‐kinase
PLAT: tissue plasminogen activator
PTCH: patched
SDS–PAGE: sodium dodecyl sulfate polyacrylamide gel electrophoresis
shRNA: short hairpin RNA
SMO: smoothened
SMOi: smoothened inhibitor
STAT3: signal transducer and activator of transcription‐3
TAM: tamoxifen
TYK2: tyrosine kinase‐2

Basal cell carcinoma (BCC) is the most common cancer in the Western world with an annual incidence of 3–4 million new cases in the US alone.^1^ Genetic activation of the hedgehog (HH)/GLI pathway by inactivating mutations in the *patched (PTCH)* gene or—more rarely—by activating mutations in the *smoothened (SMO)* gene represents the main driver signal in BCC pathogenesis. HH‐mediated hyperactivation of the zinc finger transcription factors GLI1 and GLI2 results in a malignant expression profile driving tumor growth (Fig. 1 *a*).^2^, ^3^, ^4^, ^5^, ^6^

**Figure 1:**
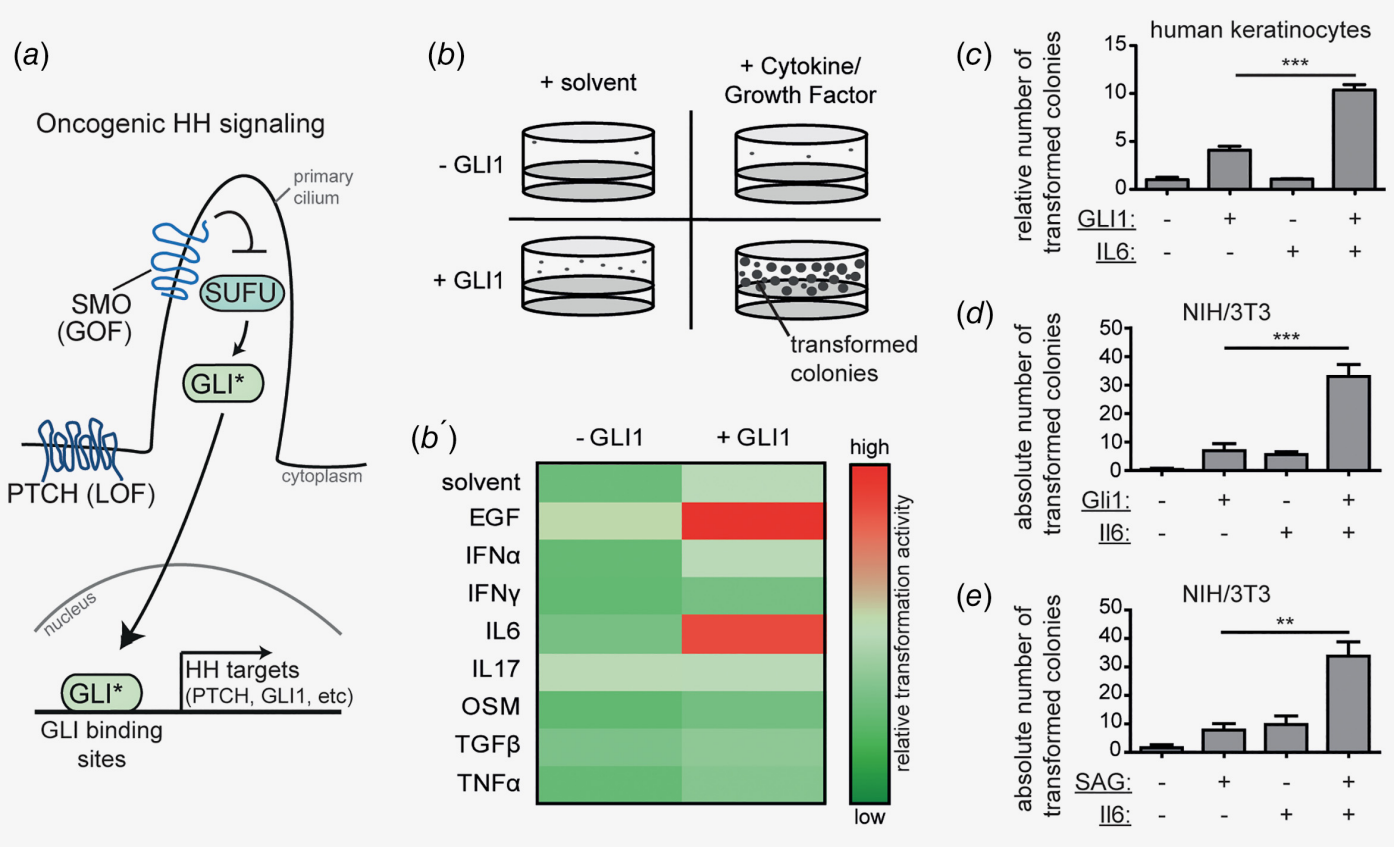
IL6 synergizes with HH/GLI signaling in oncogenic transformation.(*a*) Schematic illustration of linear, canonical HH/GLI signaling in the absence of signal cross‐talk. Loss‐of‐function mutations (LOF) in *patched (PTCH)* or gain‐of‐function mutations (GOF) in *smoothened (SMO)* account for the majority of BCC by releasing the GLI zinc‐finger transcription factors from their inhibitor suppressor of fused (SUFU). Nuclear translocation of GLI activator forms (GLI*) leads to the onset of transcriptional activation of HH/GLI target genes.(*b*) Scheme of screen for oncogenic HH modifiers. Nontumorigenic, human HaCaT keratinocytes were grown in *in vitro* transformation assays and four conditions were tested: cells were either left untreated and served as solvent‐only control (+solvent;‐GLI1), treated with cytokines or growth factors (+cytokine/growth factor;−GLI1), expressed GLI1 (+solvent;+GLI1) or a combination of both (+cytokine/growth factor;+GLI1). The number of transformed colonies served as readout. (*b*′) Heat‐map analysis of the *in vitro* screen for oncogenic HH modifiers. Changes in spheroid numbers are depicted relative to GLI1‐expressing cells treated with solvent only (+solvent;+GLI1). Red color indicates a synergistic increase in the number of transformed colonies.(*c*) Quantitative results of *in vitro* transformation assays of human HaCaT keratinocytes after GLI1 activation in combination with or without IL6 treatment.(*d*) Quantitative results of *in vitro* transformation assay of Gli1 expressing mouse NIH/3T3 cells with or without Il6 treatment as indicated. Empty vector not expressing Gli1 served as control.(*e*) Quantitative results of *in vitro* transformation assay of SAG‐responsive NIH/3 T3 cells upon SAG (100 nM) with or without Il6 stimulation as indicated.Statistical analysis by Student's *t* test; ***p < 0.001; **p < 0.01.

Small‐molecule SMO inhibitors (SMOi) show striking therapeutic efficacy in patients with advanced and metastatic BCC,^7^, ^8^ though development of drug resistance and severe adverse effects leave many patients without proper treatment options.^9^, ^10^, ^11^ Furthermore, noncanonical, SMO‐independent GLI activation has been identified as critical factor contributing to the growth of malignant cells refractory to SMOi treatment (reviewed in Refs. 12, 13, 14, 15). Therefore, understanding the intricate molecular basis and genetic landscape of HH/GLI‐driven skin cancer,^16^ including microenvironmental cues and interactions with the immune system, is key to the development of improved targeted therapies, particularly for BCC patients with *a priori* or acquired resistance to SMOi.

Inflammatory signals activated in cancer tissues are potent promoters of tumor initiation, progression and metastasis. Tumor‐promoting inflammation is often mediated by the production of proinflammatory cytokines such as interleukin‐6 (IL6) (reviewed in Refs. 17, 18). IL6 signaling is triggered by ligand binding to the high‐affinity IL6 receptor alpha (IL6R) subunit, which together with gp130 receptor subunits transduces the signal to the Janus tyrosine kinases (JAK1, JAK2 and TYK2). Upon JAK‐mediated tyrosine phosphorylation of signal transduction and activator of transcription (STAT)‐3, phospho‐STAT3 (pSTAT3) dimerizes and translocates into the nucleus, where it engages its transcriptional regulatory function. STAT3 is the main transcription factor through which IL6 signals, although IL6 can lead also to the activation of MEK/ERK, PI3K/AKT or NFkB signaling.^17^, ^19^ IL6 can also be bound by a soluble form of the IL6R followed by subsequent interaction with gp130. This so‐called IL6 trans‐signaling allows IL6 to target cells not expressing the membrane‐bound IL6R.^20^ The therapeutic relevance of IL6 signaling in malignant development is currently evaluated in a number of clinical trials with antibodies and small‐molecule inhibitors targeting oncogenic IL6/STAT3 signaling.^17^, ^21^

In light of the pivotal role of immunomodulatory cytokines and growth factors in the development and progression of malignancies, we performed in this study a candidate‐based screen to identify possible enhancers of oncogenic HH/GLI signaling in the context of BCC development. We identified the proinflammatory IL6 pathway as a novel oncogenic cooperation partner of HH/GLI in BCC and show that HH/GLI and IL6/STAT3 signaling interact at the level of *cis*‐regulatory elements of common HH‐IL6 target genes. Using conditional genetic mouse models of BCC, we demonstrate that IL6 signaling is required for the formation of HH/GLI‐driven BCC *in vivo* by synergistically promoting the proliferative effect of oncogenic HH/GLI signaling. Our study provides a rationale for combined inhibition of HH/GLI and IL6/STAT3 signaling for improved targeted therapy of BCC.

## Material and Methods

### Cell culture and treatments

Doxycycline (Dox)‐inducible GLI1‐expressing HaCaT keratinocytes and mouse BCC cell line ASZ001^22^ were grown as described previously.^23^, ^24^ Induction of GLI1 expression in HaCaT keratinocytes was done as reported in Refs. 25, 26 Murine NIH/3 T3 cells (AMS Biotechnology Ltd, Abingdon, UK) transduced with pBabe‐puro‐GLI1 or empty control vector were grown in Dulbecco's modified Eagle medium (DMEM) (Sigma, St. Louis, MO) supplemented with 10% calf bovine serum (Sigma) and penicillin–streptomycin (Sigma). Chemicals and reagents used for cell treatments are listed in Supporting Information, Table S1. Recombinant human and mouse IL6 and Dox were used at a concentration of 50 ng/ml, unless indicated otherwise. For three‐dimensional (3D) cultures, 1 × 10^4^ human HaCaT keratinocytes were seeded in 12‐well plates (Greiner Bio‐one, Kremsmünster, Austria), cultured and analyzed in a blinded fashion as described previously.^25^

### qPCR and Western blot analysis

RNA isolation, cDNA synthesis and qPCR analysis of mRNA expression were carried as described previously.^23^ qPCR analysis was done on a Rotor‐Gene Q (Qiagen, Hilden, Germany) using GoTaq qPCR Master Mix (Promega, Madison, WI). The sequences of primers used for amplification are listed in Supporting Information, Table S2. SDS‐PAGE and Western blotting were performed according to standard protocols. Applied antibodies are listed in Supporting Information, Table S3.

### RNA interference and lentiviral transduction

RNA interference and lentiviral transduction experiments were performed as described in Ref. 23. The following short hairpin RNA (shRNA) constructs selected from the Mission TRC shRNA library (Sigma) were used: shRNA IL6R#1 (TRCN0000378748), shRNA IL6R#2 (TRCN0000058780), shRNA JAK2#1 (TRCN0000003180), shRNA JAK2#2 (TRCN0000003181), shRNA STAT3#1 (TRCN0000071456), shRNA STAT3#2 (TRCN0000020843) and scrambled control shRNA (SHC002). Transduced cells were selected for puromycin resistance prior to further analysis.

### Analysis of cell proliferation

Proliferation of human HaCaT keratinocytes with Dox‐inducible GLI1 expression was analyzed with the Click‐iT® Plus EdU Alexa Fluor® 555 Imaging Kit (Thermo Fisher Scientific, Waltham, MA). Cells were treated for 72 hr with Dox, IL6 and 1 μM panJAK‐Inh I. EdU proliferation assay was performed according to the manufacturer's instructions with the following modifications: Cells were incubated with 5 μM EdU for 3 hr. Cells were stained with Alexa Fluor® picolyl acid 555 and Hoechst® 33342, and counted in a blinded fashion. The ratio of proliferative cells to Hoechst‐positive cells was calculated.

### Transgenic mice and allograft experiments

*K14creER* ^*T*^ *;Ptch* ^*fl/fl*^ *;Il6ra* ^*fl/fl*^ mice: *K14CreER* ^*T*^ (#5107), *Ptch* ^*f/f*^ (#12457) and *Il6ra* ^*f/f*^ (#12944) mice were genotyped according to the supplier's instructions (The Jackson Laboratory, Bar Harbor, ME). Tamoxifen (TAM) (Sigma) was dissolved in sunflower oil and administered at 1 mg/day by oral gavage to induce activation of Cre recombinase and knock‐out of floxed alleles. All mice were treated with TAM on postnatal days p21–25, p46, p48 and p50 to achieve efficient recombination and euthanized on day p67, when the overall health condition of the mice declined and an established phenotype was observed (Supporting Information, Figs. S5 *c* and S5 *d*).

For *in vivo* tumor growth studies, 1 × 10^6^ ASZ001 BCC cells with Stat3 knockdown (shStat3#1) or scrambled control shRNA were mixed with 25% Matrigel (BD Biosciences, San Jose, CA) and injected subcutaneously into nude mice (Charles River Laboratories, Wilmington, MA). Tumor volume was measured with a caliper and calculated according to the formula [4/3 × *π* × (length/2) × (width/2) × (height/2)].

### Histology and immunohistochemistry

Immunohistochemistry (IHC) was performed with formalin‐fixed paraffin‐embedded tissue using standard protocols and antibodies listed in Supporting Information, Table S3. Antibody detection was done using IDetect Super Stain System (IDlabs Biotechnology, Empire Genomics, Buffalo, NY). Staining was visualized using 3‐amino‐9‐ethylcarbazole (IDlabs Biotechnology) under visual control. All images were taken with a Zeiss AxioImager Z1, and quantification was performed with HistoQuest (TissueGnostics, Vienna, Austria). For tumor area determination, at least three different high‐power fields per mouse of H&E‐stained dorsal skin were analyzed and quantified. The tumor area of *Ptch‐*deficient BCC mice with functional Il6ra was set to 100%.

### Microarray analysis and GSEA

Genome‐wide mRNA expression profiling was performed on a bead array technology platform (Illumina Inc., San Diego, CA). RNA of human HaCaT keratinocytes either expressing GLI1 (Dox treatment), treated with IL6 or stimulated with a combination of both was analyzed in comparison to untreated control cells. Gene set enrichment analysis (GSEA) was performed using GSEA software v3.0 (Broad Institute of MIT and Harvard, (http://software.broadinstitute.org/gsea/).^27^ For the identification of synergistically regulated HH‐IL6 target genes, data obtained from microarray analysis were verified by qPCR analysis and the synergy score according to McMurray et al. was calculated.^28^ Synergy scores ≤0.9 defined target genes as synergistically induced in response to combined HH‐IL6 stimulation.

### Promoter and histone modification studies

*In silico* prediction of putative GLI binding sites was done using the D‐Light Software^29^ (genome sequence GRCh37/hg19) trained with the GLI binding site matrix according to Winklmayr et al.^30^ The ENCyclopedia Of DNA Elements (ENCODE) Project^31^ was used to check for STAT3 binding regions. Luciferase reporter assays and site directed mutagenesis were carried out as described previously.^23^ All constructs were confirmed by sequencing.

Chromatin‐immunoprecipitation (ChIP) assays were carried out as described previously.^32^ A total of 10 μg cross‐linked chromatin was precipitated with antibodies listed in Supporting Information, Table S3. Immunoprecipitated DNA was analyzed by qPCR on a Rotor‐Gene Q (Qiagen) using GoTaq qPCR Master Mix reagent (Promega) with primers listed in Supporting Information, Table S2. The amount of immunoprecipitated DNA in each sample was calculated by the Percent Input Method according to the manufacturer's instructions (Cell Signaling Technology, Boston, MA).

### Quantitative methylation analysis by bisulfite pyrosequencing

Methylation status of a total of 9 CpG sites in adjacent GLI and STAT3 binding site regions of human EDN2 (NM_001956) was analyzed by bisulfite pyrosequencing. Genomic DNA (500 ng) was bisulfite‐treated using the EZ DNA Methylation Kit (Zymo Research, Irvine, CA) according to manufacturer's instructions. Bisulfite‐converted DNA was PCR amplified using HotStar Taq Polymerase (Qiagen) with the primers listed in Supporting Information, Table S4. GLI binding sites were biotin‐tagged with a universal sequence (see Supporting Information, Fig. S4 *f*). Pyrosequencing was performed on the PyroMark Q24 Advanced System (Qiagen).

### Statistical analysis

Significant differences between two groups were determined using a two‐tailed, unpaired *t* test. *p* values of <0.05 were assigned significance and *p* values are considered as follows: *p < 0.05, **p < 0.01 and ***p < 0.001. All values are given as means ±standard error of the mean (s.e.m.) and were analyzed by GraphPad Prism® 7 (GraphPad Software, San Diego, CA). Numbers of animals are stated in the respective figure legends.

### Ethics

Human BCC tissue arrays for immunohistochemistry analyses were used in accordance with the guidelines of the Austrian ethics committee application (EK405/2006, extension 11/10/2016). Animal experiments and care were carried out in accordance with the guidelines of institutional authorities and approved by the Federal Ministry of Science, Research and Economy (BMWF‐66.012/0017‐II/3b/2012, BMWFW‐66.012/0016‐WF/V/3b/2015).

## Results

### IL6 synergizes with HH/GLI in oncogenic transformation

To screen for immunomodulatory cytokines and/or growth factors able to cooperate with HH/GLI signaling (Fig 1 *a*) in oncogenic transformation, we used nontumorigenic, human keratinocytes (HaCaT) with doxycycline‐inducible GLI1 expression.^24^ Importantly, GLI1 expressing keratinocytes do not display a fully transformed phenotype but require additional cooperative signals for malignant growth.^25^ We took advantage of this characteristic and performed a candidate‐based *in vitro* transformation screen for immunomodulatory cytokines and growth factors that are able to cooperate with HH/GLI in the process of oncogenic transformation. As readout for oncogenic transformation we monitored clonal growth in 3D anchorage‐independent settings, which we have previously shown to correlate with *in vivo* tumor growth (Fig 1 *b*).^25^ In total, we have screened 13 secreted factors, of which seven factors passed the preselection criteria, including detectable expression of the cognate receptor as judged by RNAseq data (Human Protein Atlas http://www.proteinatlas.org) 33 and the ability to induce the activation of established downstream effectors (Supporting Information, Table S5). As positive control for the integrity of the screen, we monitored cellular transformation by concomitant epidermal growth factor (EGF) signaling and GLI1 expression, which we have previously shown to have potent synergistic transformation capacity (Fig 1 *b′*).^25^ As shown in Figures 1 *b′* and 1*c*, GLI1 expression and simultaneous treatment with the proinflammatory cytokine IL6 resulted in a synergistic increase in the number of transformed colonies in 3D anchorage‐independent assays compared to single treatments. Neither of the other soluble signaling factors tested was able to enhance the oncogenic transformation efficiency of GLI1. We also observed synergistic transformation of Hh‐responsive mouse NIH/3T3 fibroblasts upon combined Gli1 expression and Il6 treatment (Fig. 1 *d*) and upon combined treatment with the HH pathway activator smoothened agonist (SAG)^34^ and Il6 (Fig. 1 *e*). Together, these data identify IL6 signaling as a novel synergistic interaction partner of HH/GLI in oncogenic transformation.

### IL6 signals through IL6R/JAK2/STAT3 to cooperate with oncogenic HH/GLI signaling

**Figure 2:**
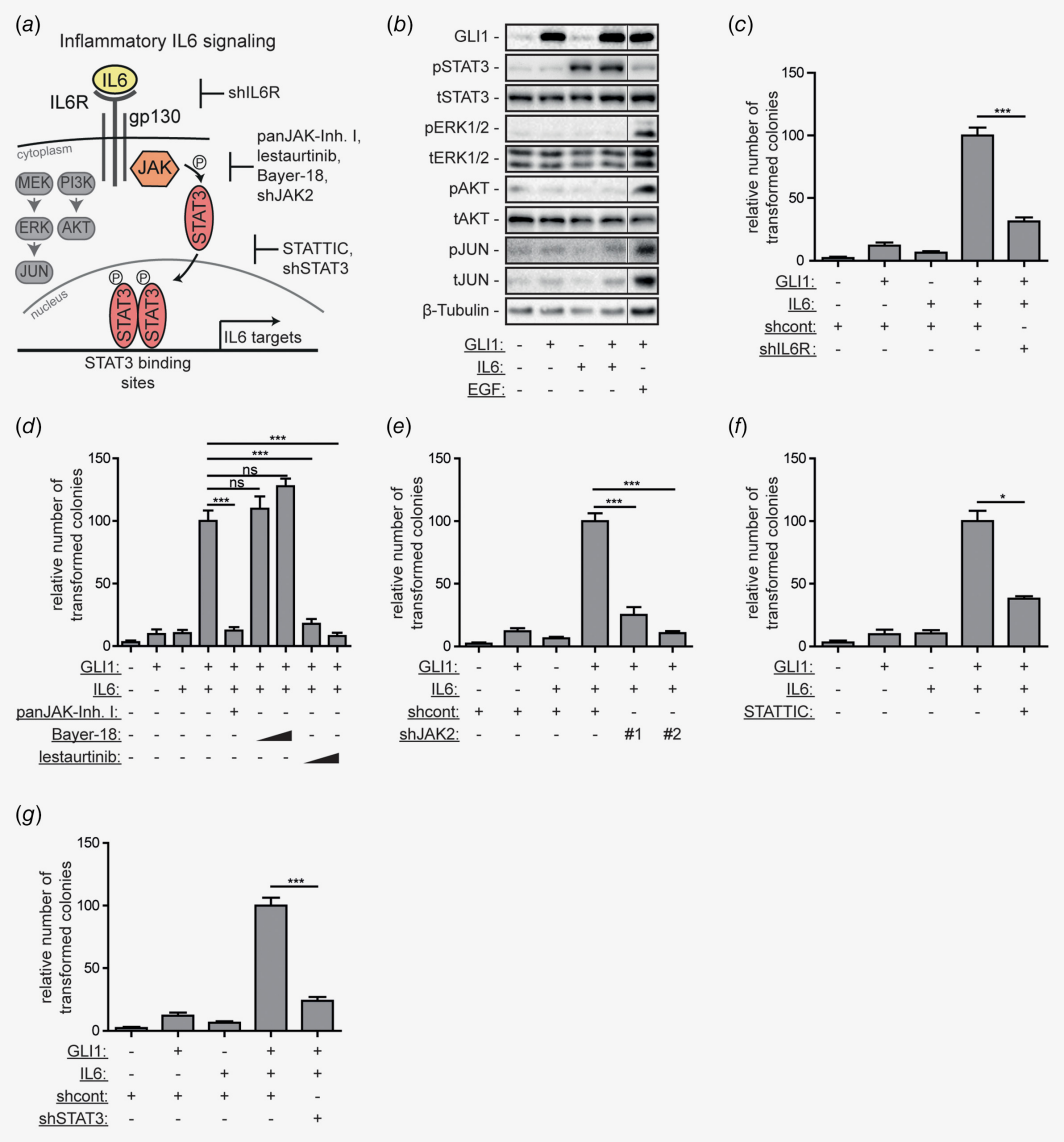
IL6/JAK2/STAT3 signaling cooperates with HH/GLI in oncogenic transformation.(*a*) Illustration of IL6 signaling and downstream pathway activation. Binding of IL6 to its receptor can activate at least three downstream signaling cascades: JAK/STAT3, MEK/ERK/JUN and PI3K/AKT signaling. In the context of malignant transformation, IL6 induces JAK/STAT3 activation. The genetic and pharmacologic approaches to inhibit IL6 signaling effectors are depicted.(*b*) Western blot analysis of GLI1 expressing human HaCaT keratinocytes treated with IL6, or 10 ng/ml EGF. β‐tubulin served as loading control. Fine black lines indicate cropping of intermediate lanes from the same Western blots. p, phospho; t, total;(*c*–*g*) Quantitative analysis of *in vitro* transformation assays using HaCaT keratinocytes. Cells were treated either with solvent, Dox to induce GLI1, IL6 or Dox and IL6. Additionally, double‐stimulated cells (+GLI1;+IL6) were treated as follows: (*c*) with shRNA against IL6R (shIL6R, shRNA #1 in Supporting Information, Fig. S1 *a*), (*d*) with panJAK‐Inh I (1 μM), Bayer‐18 (100 nM and 300 nM) or lestaurtinib (100 nM and 300 nM), (*e*) with shRNA constructs against JAK2 (shJAK2#1, shJAK2#2), (*f*) with STATTIC (1 μM) or (*g*) with shRNA against STAT3 (shSTAT3). ns, not significant; shcont, scrambled nontarget control shRNA; Statistical analysis by Student's *t* test; ***p < 0.001; *p < 0.05.

Next, we aimed to identify the IL6‐induced downstream signaling cascade that integrates with HH/GLI in the synergistic transformation of epidermal cells. IL6 can signal through the activation of MEK/ERK, PI3K/AKT or JAK/STAT3, the latter representing the canonical intracellular signal relay mechanism (Fig. 2 *a*). As shown in Figure 2 *b* and Supporting Information, Figure S1 *b*, IL6 treatment of human HaCaT keratinocytes resulted in the JAK‐dependent phosphorylation of STAT3 (pSTAT3) but failed to activate PI3K/AKT and MEK/ERK signaling. This clearly differentiates the mechanism of HH‐IL6 synergy from the previously described synergism of HH/GLI and EGFR signaling, which involves MEK/ERK/JUN activation downstream of EGFR.^25^

To identify the respective IL6 signal effectors in HH‐IL6‐mediated oncogenic transformation, we performed systematic pharmacologic and genetic inhibition experiments (see overview in Fig. 2 *a* and Supporting Information, Fig. S1 for validation of the functionality of inhibitors and short hairpin RNAs (shRNAs)). As shown in Figure 2 *c*, shRNA‐mediated knockdown of the receptor subunit IL6R (shIL6R) prevented synergistic transformation of human HaCaT keratinocytes in response to combined activation of IL6 and HH/GLI signaling. In line with the protein data shown in Figure 2 *b*, treatment with panJAK‐Inh I resulted in a significant reduction of synergistic oncogenic transformation in response to combined IL6‐GLI1 activation (Fig. 2 *d*). To identify the respective signal‐mediating JAK involved in the HH‐IL6 cooperation, we first targeted TYK2 and JAK2 using selective kinase inhibitors Bayer‐18 and lestaurtinib, respectively. Treatment with the TYK2 inhibitor Bayer‐18 did not abrogate the transformed phenotype, whereas the JAK2 inhibitor lestaurtinib efficiently prevented colony formation (Fig. 2 *d*). We corroborated the essential role of JAK2 by genetic targeting with two independent shRNAs (shJAK2#1 and shJAK2#2). In line with chemical JAK2 perturbation, knockdown of JAK2 significantly diminished HH‐IL6‐dependent oncogenic transformation (Fig. 2 *e*). To address the involvement of STAT3 downstream of IL6/JAK2, we blocked STAT3 pharmacologically and genetically. The small‐molecule STAT3 inhibitor STATTIC^35^ (Fig. 2 *f*) and depletion of STAT3 expression using shSTAT3 (Fig. 2 *g*) both resulted in a significant reduction of HH‐IL6 induced transformation. Consistently, also treatment of Il6‐stimulated/Gli1 expressing NIH/3T3 cells (Supporting Information, Fig. S2 *a*) or Il6/SAG‐stimulated NIH/3T3 cells (Supporting Information, Fig S2 *b*) with panJAK‐Inh I or lestaurtinib impaired Hh‐Il6 driven oncogenic transformation. Taken together, our data show that oncogenic HH‐IL6 signal cooperation requires activation of the IL6R/JAK2/STAT3 signaling cascade.

### HH/GLI‐IL6/STAT3 cross‐talk cooperatively regulates gene expression by signal integration at the level of *cis*‐regulatory elements of common HH‐IL6 target genes

**Figure 3:**
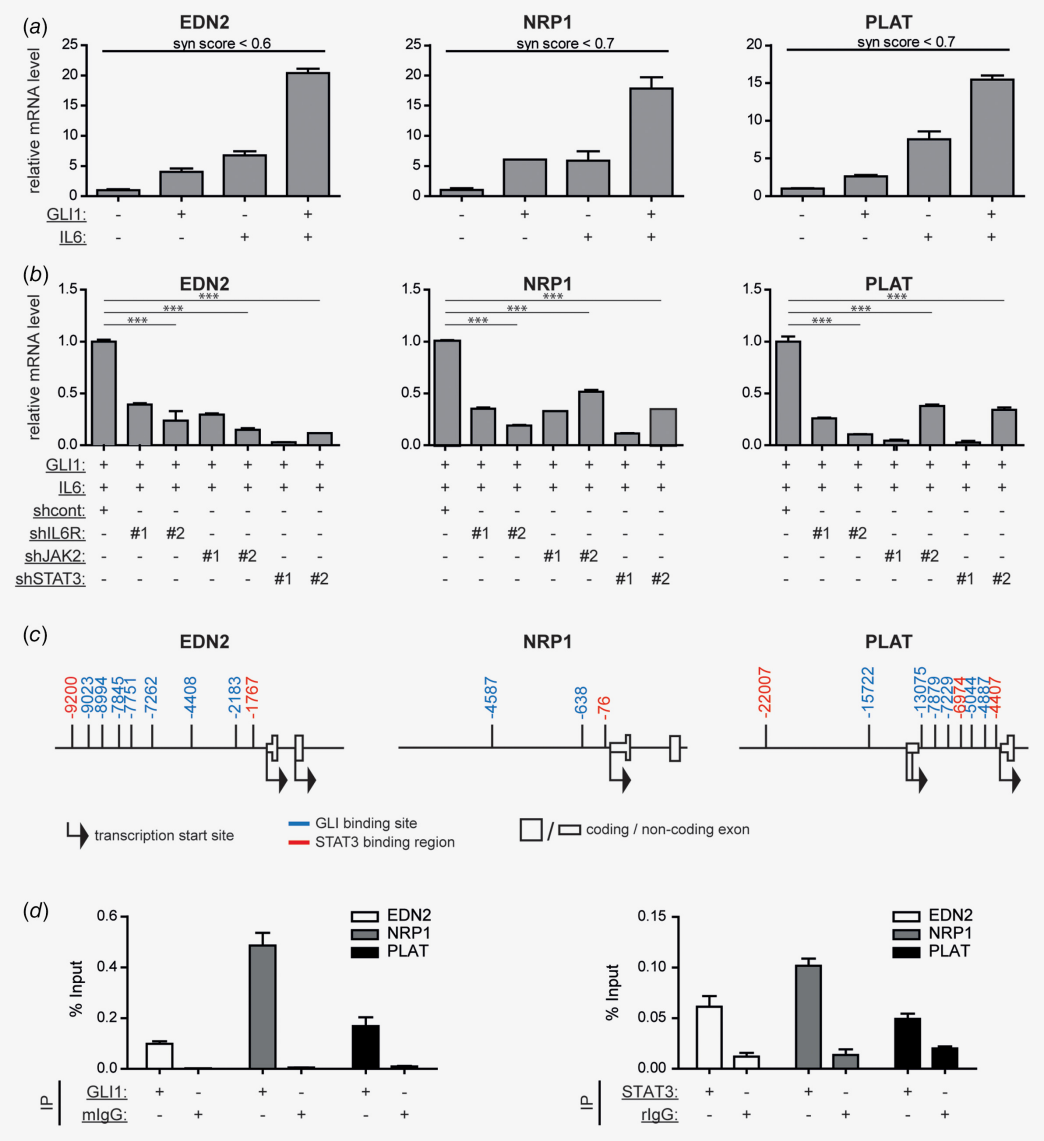
Integration of HH‐IL6 signaling at *cis*‐regulatory regions of common HH‐IL6 target genes.(*a*) mRNA expression analysis by qPCR of selected HH‐IL6 target genes (EDN2, NRP1, PLAT) in human HaCaT keratinocytes in response to Dox‐induced GLI1 expression, IL6 treatment or a combination of both. Synergy (syn) score values of ≤0.9 indicate synergistic cooperation of simultaneous HH‐IL6 signaling.(*b*) qPCR mRNA expression analysis of HH‐IL6 target genes in human HaCaT keratinocytes in response to GLI1 expression, IL6 stimulation and additional knockdown of IL6R (shIL6R#1, shIL6R#2), JAK2 (shJAK2#1, shJAK2#2) or STAT3 (shSTAT3#1, shSTAT3#2). Signals are relative to double‐stimulated cells transduced with shcont non‐target shRNA. shcont, scrambled nontarget control shRNA; ***p < 0.001;(*c*) *In silico* analysis of the *cis*‐regulatory region of selected HH‐IL6 target genes (EDN2, NRP1, PLAT) for the presence of STAT3 binding regions and putative GLI binding sites. Numbers show the start position of GLI binding sites (blue) and STAT3 binding regions (red) relative to the transcriptional start site (TSS).(*d*) ChIP analysis of selected HH‐IL6 target genes (EDN2, NRP1, PLAT) for GLI1 (left) and STAT3 binding (right). Human HaCaT keratinocytes expressing Dox‐inducible MYC‐tagged GLI1 treated with IL6 were analyzed. Mouse IgG (mIgG) or rabbit IgG (rIgG) served as negative controls.

Next, we aimed to decipher the molecular mechanisms underlying oncogenic HH‐IL6 signal cooperation, first by testing for possible reciprocal modifications of signaling activities at multiple regulatory levels. We examined if STAT3 modifies GLI1 protein stability, expression or nuclear localization, but found no evidence for that (Supporting Information, Figs. S3 *a*–S3 *d*). Vice versa, GLI1 expression neither affected the intracellular localization of STAT3 (Supporting Information, Fig. S3 *d*) nor STAT3 activation in response to IL6 signaling (Supporting Information, Fig. S3 *e*). We therefore hypothesized that cell transformation induced by cooperating oncogenic signals is caused by synergistic modulation of gene expression via signal integration at the level of *cis*‐regulatory regions of common HH‐IL6 target genes, analogous to our previous findings of HH‐EGFR signal cooperation.^23^ To test this hypothesis, we performed Illumina bead array‐based transcriptomics of human HaCaT keratinocytes with either active HH/GLI1, IL6/STAT3 or a combination of both. We identified genes synergistically regulated by combined HH‐IL6 signaling, where synergistic regulation was defined by synergy score values of ≤0.9 (see Ref. 28). From the list of synergistically regulated HH‐IL6 target genes we selected endothelin 2 (EDN2), neuropilin 1 (NRP1) and tissue‐type plasminogen activator (PLAT) as representative genes highly expressed in BCC (Supporting Information, Fig. S4 *a*)^36^ and used these HH‐IL6 regulated genes as molecular readout to decipher the mechanisms of signal integration (Fig. 3 *a*). Intriguingly, we found that transcriptional activation of the known HH/GLI target gene PTCH was unaffected by simultaneous IL6 signaling (Supporting Information, Fig. S4 *b*), suggesting that IL6 does not simply boost the expression of HH/GLI target genes but—in combination with HH/GLI—selectively activates a distinct set of common HH‐IL6 targets. Having shown that oncogenic transformation induced by combined HH‐IL6 signaling depends on activation of IL6R/JAK2/STAT3 signaling activity, we first tested whether synergistic HH‐IL6 target gene regulation also involves these IL6 effectors. In line with our data on oncogenic transformation by combined HH‐IL6 signaling, RNAi‐mediated inhibition of IL6R, JAK2 or STAT3 effectively abrogated the expression of the HH‐IL6 target genes EDN2, NRP1 and PLAT (Fig. 3 *b*).

We next tested the hypothesis that selective activation of HH‐IL6 target genes involves co‐binding of GLI1 and STAT3 transcription factors to the *cis*‐regulatory region of HH‐IL6 targets. Interestingly and as depicted in Figure 3 *c*, all three HH‐IL6 target genes harbor putative GLI and STAT3 binding sites in close proximity as predicted by bioinformatics analysis. By contrast, *in silico* analysis of the PTCH promoter revealed GLI binding sites 30, but failed to identify STAT3 binding sites (Supporting Information, Fig. S4 *c*), consistent with PTCH expression being insensitive to IL6 signaling. To analyze binding of GLI1 and STAT3 to HH‐IL6 target gene promoters, we performed chromatin immunoprecipitation (ChIP) of GLI1 and STAT3 and found that both transcription factors bind to the predicted binding sites in the representative HH‐IL6 target genes (Fig. 3 *d*).

To corroborate the ChIP data, we cloned the promoter region of the HH‐IL6 target PLAT and performed luciferase reporter assays to analyze the functionality of the GLI and STAT3 binding sites in the respective *cis*‐regulatory region. Site directed single and combined mutagenesis of the GLI and STAT3 binding sites in the PLAT promoter confirmed the requirement of both binding sites for full‐blown promoter activation (Supporting Information, Fig. S4 *d*).

Furthermore, we analyzed possible epigenetic changes triggered by combined HH‐IL6 signaling. ChIP analysis of activating histone modifications revealed an increase in H3K27 acetylation upon combined HH/GLI and IL6/STAT3 activity when compared to single pathway activity (Supporting Information, Fig. S4 *e*). As combined HH/GLI‐IL6/STAT3 signaling did not affect the DNA methylation status (see Supporting Information, Fig. S4 *f*), we conclude that cooperation of GLI and STAT3 does not depend on changes in CpG methylation.

Together, our data suggest a mechanistic model, where HH‐IL6 signal integration involves simultaneous binding of HH‐induced GLI and IL6‐induced STAT3 transcription factors to the *cis*‐regulatory region of common HH‐IL6 target genes.

### IL6 signaling is required for *in vivo* growth of HH/GLI‐driven BCC

**Figure 4:**
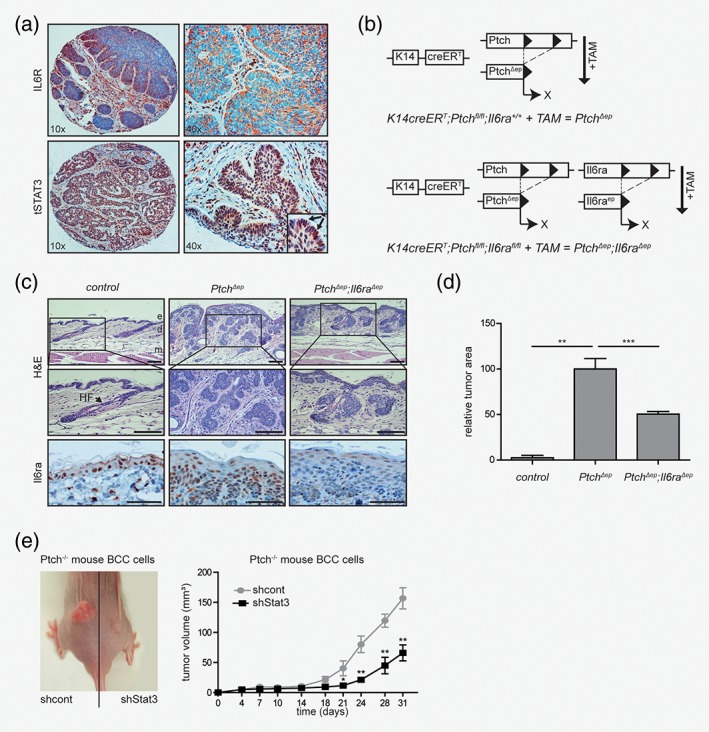
IL6 signaling is required for *in vivo* growth of HH‐driven BCC lesions.(*a*) Representative IHC staining of IL6R and tSTAT3 in human nodular BCC (*n*_(samples analyzed)_ = 16). Arrows mark nuclear STAT3.(*b*) Illustration of the genetic approach for conditional depletion of *Ptch* and *Il6ra* under the control of the epidermis‐specific K14 promoter.(*c*) Representative hematoxylin–eosin (H&E) staining and immunohistochemistry (IHC) staining of Il6ra in dorsal skin sections of mice with the indicated genotype. Il6ra expression in patched‐deficient (*Ptch* ^*Δep*^) epidermis is also shown in Supporting Information, Fig S5 *a*. e, epidermis; d, dermis; m, muscle; HF, hair follicle; scale bars (H&E), 50 μm; scale bars (Il6ra), 25 μm;(*d*) Quantitative analysis of tumor area of control mice (*n* = 2), *Ptch* ^*Δep*^ mice (*n* = 6) and *Ptch* ^*Δep*^ *;Il6ra* ^*Δep*^ mice (*n* = 8) relative to tumor load of *Ptch* ^*Δep*^ mice. Control mice occasionally developed small BCC (due to leakiness of the used Cre‐deleter strain) and were used as basal level for tumor area analysis.(*e*) Engraftment of murine Ptch‐deficient BCC cells (ASZ001) with shRNA‐mediated knockdown of Stat3. Left panel: control cells transduced with scrambled nontarget control shRNA (shcont) were grafted subcutaneously into the left and Stat3 knockdown cells (shStat3) into the right lower flank of nude mice, respectively. Right panel: quantitative analysis of tumor growth in nude mice (*n* = 7). Tumor growth was measured over a period of 31 days.Statistical analysis by Student's *t* test: ***p < 0.001; **p < 0.01, *p < 0.05.

We next addressed the *in vivo* relevance of HH‐IL6 synergism in HH/GLI‐driven BCC. As a first approach, we analyzed by immunohistochemistry (IHC) human and murine BCC for expression of IL6 effectors such as IL6R and STAT3. In line with a putative oncogenic role of IL6 signaling in HH/GLI‐driven BCC, we found expression of both proteins in human and mouse BCC tissue (Fig. 4 *a* and Supporting Information, Fig. S5 *a*). Of note, human BCC display prominent IL6R expression on the cell surface of palisading BCC cells. Consistently, we also detected active, nuclear STAT3 staining in this tumor area. In addition, marked expression of Il6ra and nuclear Stat3 was detected in mouse BCC‐like lesions (Supporting Information, Fig. S5 *a* and Supporting Information), supporting a crucial role for IL6 signaling in HH‐induced skin carcinogenesis.

To address a possible functional contribution of IL6 signaling to HH/GLI‐driven BCC, we genetically inactivated Il6 signaling in a conditional mouse model of human BCC. For this purpose, we crossed *Keratin14creER* ^*T*^ *;Ptch* ^*fl/fl*^ (Ptch^*Δep*^) mice, which develop BCC lesions upon tamoxifen (TAM)‐induced, epidermal‐specific *Ptch* deletion,^37^ with mice harboring a conditional, floxed *Il6ra* allele (*Il6ra* ^*fl/fl*^)^38^ to generate BCC mice with an additional tumor‐specific deletion of *Il6ra* (*Ptch* ^*Δep*^ *;Il6ra* ^*Δep*^) (Fig. 4 *b*). As shown in Figure 4 *c*, Ptch^*Δep*^;*Il6ra* ^*+/+*^ mice developed numerous BCC‐like lesions. Intriguingly, mice with concomitant deletion of *Il6ra* (*Ptch* ^*Δep*^ *;Il6ra* ^*Δep*^) presented significantly smaller lesions compared to *Il6ra*‐proficient mice (Fig. 4 *c*). Quantification of tumors grown revealed a 50% reduction of the relative tumor area in the epidermis of *Ptch* ^*Δep*^ *;Il6ra* ^*Δep*^ mice compared to *Ptch* ^*Δep*^;*Il6ra* ^*+/+*^ controls (Fig. 4 *d*).

To also address the role of STAT3 in HH/GLI‐driven BCC growth, we depleted by RNAi the expression of Stat3 in murine Ptch‐deficient BCC cells and compared the *in vivo* growth of Stat3‐deficient BCC cells with that of Stat3‐proficient controls. As shown in Figure 4 *e*, shRNA‐mediated knockdown of Stat3 expression significantly reduced the *in vivo* growth of murine BCC cells (Fig. 4 *e* and Supporting Information, Fig. S5 *b*).

Together with our *in vitro* studies on oncogenic transformation, these data suggest that IL6/STAT3 cooperates with HH/GLI signaling to promote BCC growth.

### Cooperation of HH/GLI and IL6 signaling promotes epidermal proliferation

**Figure 5:**
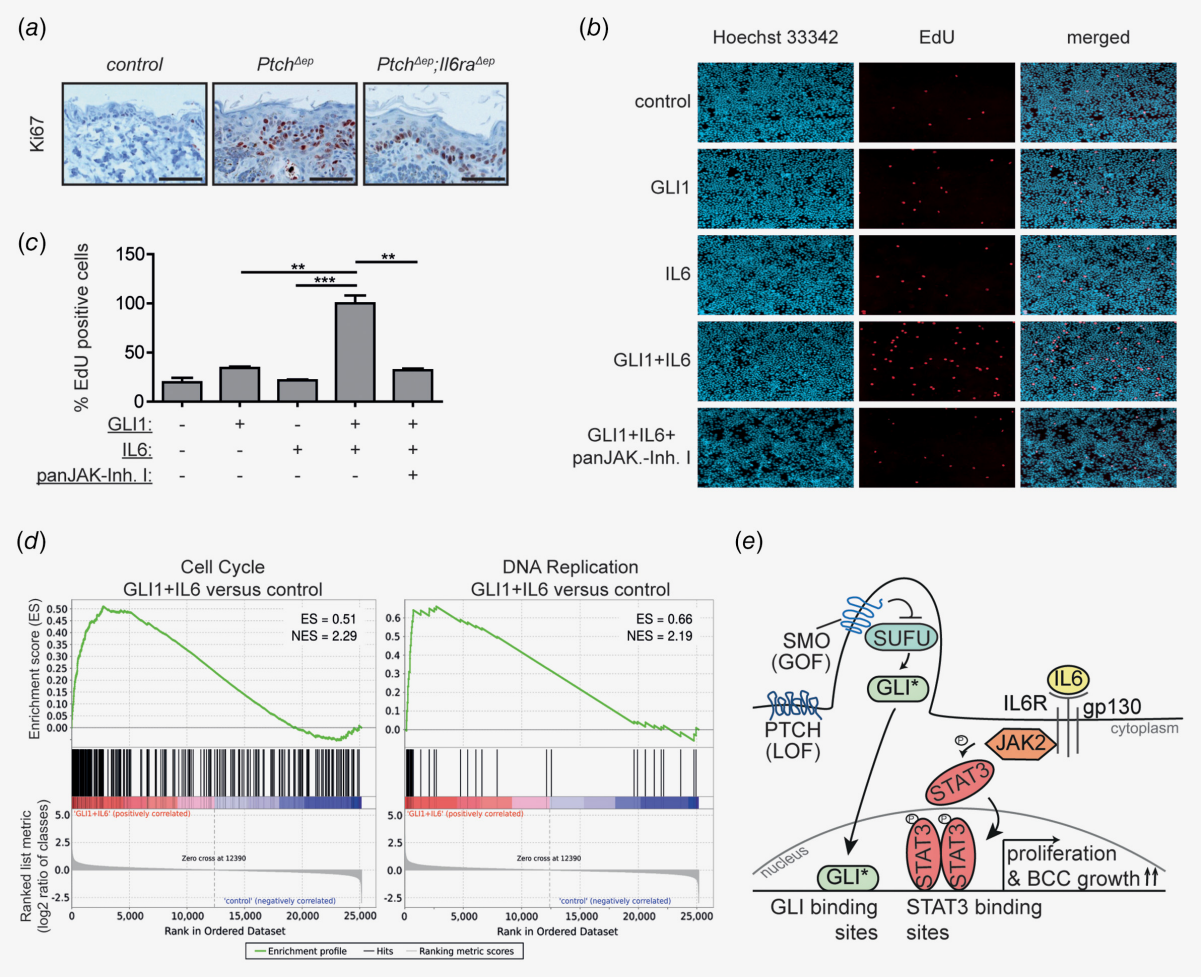
HH‐IL6 cooperation promotes epidermal proliferation.(*a*) Representative IHC staining of Ki67 in dorsal skin sections of the indicated phenotypes. Scale bars, 25 μm;(*b*) Representative fluorescence microscopy images of EdU assays. Blue, Hoechst 33342 (Cell nuclei); red, EdU‐positive, proliferating cells;(*c*) Quantitative analysis of cell proliferation in response to single and combined HH/GLI‐IL6 activity in human HaCaT keratinocytes. panJAK‐Inh I (1 μM) was used to block IL6/JAK signaling. Statistical analysis, Student's *t* test; ***p < 0.001; **p < 0.01; *p < 0.05;(*d*) Gene‐set enrichment analysis (GSEA) of cell cycle (left) and DNA replication (right) gene sets fed with genes induced by combined HH‐IL6 signaling compared to untreated controls. Genes were sorted according to their fold change in expression between keratinocytes with activated HH/GLI‐IL6 signaling and control cells on the *x*‐axis. NES, normalized enrichment score;(e) Proposed model of cooperative HH/GLI and IL6/STAT3 driving BCC growth by signal integration at the level of *cis‐*regulatory regions of common target genes via co‐occupancy of joint promoters. Binding of active GLI (GLI*) and STAT3 (pSTAT3) to their respective binding sites in shared HH‐IL6 target gene promoters synergistically enhances proliferation and BCC growth. LOF, loss‐of‐function mutation; GOF, gain‐of‐function mutation.

Based on the pronounced expression of IL6R and STAT3 in the peripheral growth zone of human BCC and the requirement of Il6ra and Stat3 for efficient *in vivo* growth of mouse BCC, we hypothesized that cooperation of IL6 and HH/GLI may promote proliferation of BCC. We therefore analyzed by IHC the skin of *Ptch* ^*Δep*^ *;Il6ra* ^*Δep*^ and *Ptch* ^*Δep*^ *;Il6ra* ^*+/+*^ mice for the proliferation marker Ki67. In BCC lesions of *Ptch* ^*Δep*^ *;Il6ra* ^*+/+*^ mice, Ki67 positive cells were distributed throughout the tumor tissue (Fig. 5 *a*). By contrast, in the dorsal skin of *Ptch* ^*Δep*^ *;Il6ra* ^*Δep*^ mice, the proliferative Ki67‐positive cells were restricted to the basal layer of the skin, similar to the normal proliferation pattern of healthy skin. To corroborate the putative proliferative role of HH/GLI and IL6 cooperation, we also performed *in vitro* proliferation studies using GLI1 expressing human HaCaT keratinocytes as a model for epidermal proliferation 24. Consistent with an enhancement of epidermal proliferation by HH‐IL6, combined activation of GLI1 and IL6 signaling significantly increased keratinocyte proliferation compared to single treatments (Figs. 5 *b* and 5*c*). Perturbation of the HH‐IL6 cooperation with the panJAK‐Inh I resulted in significantly decreased proliferation as assessed by EdU labeling. In addition, gene set enrichment analysis (GSEA) of HH‐IL6‐regulated mRNA expression revealed cell cycle and DNA replication genes to be significantly enriched in the target gene set, further supporting a proliferative role of HH‐IL6 signaling in BCC development (Fig. 5 *d*). In summary, our findings suggest a model where HH‐IL6 cooperation at the level of common target genes promotes BCC growth by enhancing tumor cell proliferation (Fig. 5 *e*).

## Discussion

Aberrant activation of HH/GLI signaling plays an etiologic role in a wide variety of human cancer entities and despite several setbacks in clinical trials, targeting HH/GLI remains a promising treatment strategy with the potential for curative effects by eradicating cancer stem cells involved in tumor initiation, metastasis and drug resistance.^14^, ^39^, ^40^, ^41^ Despite the remarkable therapeutic benefits of SMOi in BCC, development of resistance, severe adverse effects and recurrence after cessation of drug treatment^9^, ^42^, ^43^, ^44^ highlight the need for novel strategies that not only focus on HH inhibition but also take into account interacting pathways modulating the oncogenicity of HH signaling.

Although genetic and epigenetic alterations within cancer cells are the main drivers of malignant development, it has recently become clear that intricate reciprocal interactions of cancer cells with the tumor microenvironment and the immune system are pivotal for malignant progression.^18^ In this study, we performed a candidate‐based screen for immune‐related modifiers of oncogenic HH/GLI signaling and identified a striking tumor promoting role of the proinflammatory cytokine IL6 in HH/GLI‐driven oncogenic transformation and BCC development. We show that synergistic transformation by HH/GLI‐IL6 signaling relies on IL6/JAK2‐mediated activation of STAT3. In this context, it is intriguing to note that combined stimulation with Oncostatin M (OSM) and HH/GLI failed to induce transformation despite strong STAT3 activation upon OSM treatment (Fig. 1 *b′*). Whether the inability of OSM signaling to cooperate with HH/GLI is due to for instance distinct signal strength, duration, STAT heterodimerization or parallel activation of tumor suppressive processes is unclear and needs to be addressed in future studies.

Our genetic, ChIP‐ and reporter assay based analyses suggest that HH‐IL6 signal integration involves concomitant binding of IL6‐activated STAT3 and GLI activator forms such as GLI1 to the *cis*‐regulatory regions of HH‐IL6 target genes, thereby driving selective and synergistic activation of target gene expression. We have also shown that HH‐IL6 signal integration cooperatively enhances epidermal proliferation, suggesting that simultaneous HH‐IL6 signaling supports tumor growth by synergistically activating a proliferative expression profile. This is in agreement with the genetic inactivation of Il6ra and Stat3 function in murine models of BCC as well as with the respective *in situ* expression of IL6 effectors in human BCC, which together support the pathophysiological and clinical relevance of our findings. Whether the protumorigenic effect of combined HH‐IL6 signaling is directly mediated by the HH‐IL6 targets EDN2, PLAT and NRP1 requires further functional studies. In this context, it is noteworthy that PLAT and NRP1 play a well‐documented role in angiogenesis, in line with previous reports about a putative angiogenic function of IL6 in BCC.^45^, ^46^, ^47^ However, as we did not detect a decrease in CD31^+^ endothelial cells in Il6ra‐deficient mouse BCC (data not shown), it appears rather unlikely that HH‐IL6 signal integration drives BCC growth by supporting tumor angiogenesis.

This study also raises the question about the source of IL6. Paracrine IL6 signaling may emanate for instance from macrophages,^48^ though the precise immune cell status of established BCC has not yet been characterized in detail. As an alternative to paracrine IL6 signaling, activation of HH/GLI may itself stimulate the production of IL6 in the cancer cells. Indeed, we have previously shown that activation of GLI2 enhances the expression of IL6 in epidermal cells.^26^ Also, GLI1 has been shown to directly induce IL6 expression in stromal cells of pancreatic adenocarcinoma lesions, triggering paracrine STAT3 activation in the tumor cell compartment.^49^ Whether IL6 signaling is activated by oncogenic HH/GLI within the tumor cell compartment or communicated to BCC via the tumor microenvironment, infiltrating immune cells or via sIL6R‐mediated trans‐signaling remains to be addressed in future studies.

The possible therapeutic relevance of our findings is further underlined by the promising efforts to develop efficacious anti‐IL6/JAK/STAT3 drugs for the treatment of various solid and hematopoietic malignancies. Small‐molecule inhibitors or antagonistic antibodies targeting critical effectors of IL6 signaling including IL6 itself, IL6R, gp130, JAK1/JAK2 and STAT3 have recently been approved or are currently evaluated in several clinical trials with patients suffering from cancer entities with a documented involvement of HH/GLI signaling such as breast and non‐small‐cell lung cancer (for review, see Refs. 17, 21, 50 and references therein).

The identification of the IL6/JAK/STAT3 signaling cascade as cooperative partner in HH/GLI‐associated cancers provides a new rationale for evaluating combined HH‐IL6 targeting in BCC to improve the therapeutic efficacy of SMO inhibitor treatments.

## Supporting information


**Figure S1** Functionality of shRNA constructs and inhibitors. (*a*) Knockdown efficiency of shRNA against IL6R (shIL6R#1, shIL6R#2), JAK2 (shJAK2#1, shJAK2#2) and STAT3 (shSTAT3#1, shSTAT3#2) validated by qPCR analysis of mRNA levels (left panels) and by Western blot analysis (right panels) of HaCaT keratinocytes. qPCR signals are given relative to non‐target shRNA (shcont). (*b*) Western blot analysis of pSTAT3 expression in HaCaT keratinocytes treated with IL6 plus/minus panJAK‐Inh I (1 μM), lestaurtinib (300 nM, 500 nM and 1 μM) and Bayer‐18 (300 nM, 500 nM and 1 μM). (*c*) Western blot analysis of pSTAT3 levels upon stimulation with the STAT3 activating cytokine Oncostatin M (50 ng/ml) and STATTIC (0.1, 1, 10 and 100 μM). shcont, scrambled non‐target control shRNA; p, phospho; t, total; β‐Actin, β‐Tubulin and tERK served as loading controls.
**Figure S2** Perturbation of Hh‐Il6‐mediated transformation in NIH/3 T3 cells. (*a*) Quantification of *in vitro* transformation assay using Gli1 expressing mouse NIH/3 T3 cells treated with Il6, panJAK‐Inh I (1 μM) or lestaurtinib (300 nM and 500 nM) as indicated. Empty vector not expressing Gli1 served as control. (*b*) Quantitative analysis of *in vitro* transformation assay of SAG‐responsive NIH/3 T3 cells upon SAG (100 nM), Il6 or panJAK‐Inh I (1 μM) stimulation. Statistical analysis, Student's *t* test; ***p < 0.001.
**Figure S3** Lack of evidence for mutual regulation of GLI1 and STAT3. (*a*) Western blot analysis of human HaCaT keratinocytes upon Dox and IL6 treatment demonstrated no impact of active IL6/STAT3 signaling on GLI1 protein stability. Additional treatment with the proteasome inhibitor MG132 (25 μM) did not change GLI1 protein levels indicating no regulation by the proteasome machinery. Protein samples from untreated, single or combined treated cells were harvested after 18 hours treatment. (*b*) Dox‐induced GLI1 expression in human HaCaT keratinocytes is unaffected by knockdown of STAT3 (shSTAT3#1) as revealed by Western blot analysis. (*c*) Western blot analysis of GLI1 expressing human HaCaT keratinocytes showed no effect of IL6 signaling on GLI1 protein levels and time‐resolved expression analysis revealed no changes in GLI1 protein levels over time. Human HaCaT keratinocytes were pretreated with Dox for 48 hr and the time course was started by adding IL6. GLI levels were detected after 0, 6, 12, 18 and 24 hr. Control cells were included for the 0 hr timepoint. (*d*) Western blot analysis of cytosolic and nuclear proteins isolated from human HaCaT keratinocytes demonstrated no impact of IL6/STAT3 signaling on nuclear import or export of GLI1 protein. Human HaCaT keratinocytes were pretreated with Dox for 48 hr and then stimulated with IL6 for 18 hr. β‐Tubulin and PARP served as loading controls for cytoplasmic and nuclear proteins, respectively. (*e*) Human HaCaT keratinocytes with concomitant HH and IL6 signaling showed that GLI1 expression did not change tSTAT3 and pSTAT3 levels. Furthermore, IL6 treatment did not affect tSTAT3 protein levels. Human HaCaT keratinocytes were pretreated with Dox for 48 hr and then either stimulated by Dox, IL6 or a combination of both for 18 hr. p, phospho; t, total; shcont, scrambled non‐target control shRNA; β‐Actin, β‐Tubulin and PARP served as loading controls; Fine black lines indicate cropping of intermediate lanes from the same Western blots.
**Figure S4** GLI1 and STAT3 interaction at promoters of common HH‐IL6 target genes. (*a*) qPCR analysis of HH‐IL6 target gene expression (Edn2, Nrp1 and Plat) in BCC‐like lesions derived from mice with epidermal‐specific deletion of Ptch (*Ptch*D*ep*) compared to Ptch‐proficient control skin (*control*). (*b*) qPCR analysis of the canonical HH target gene PTCH. Untreated, single (GLI1 or IL6) or simultaneously treated human HaCaT keratinocytes (GLI1 + IL6) were analyzed. Synergy score > 0.9. (*c*) *In silico* analysis of the cis‐regulatory region of the HH target gene PTCH for the presence of STAT3 binding regions and putative GLI binding sites. Numbers show the relative start positions of GLI binding sites (blue) to the transcription start site (TSS). The arrow shows the Sternberg et al. HH‐IL6 signal cooperation in BCC ‐ Supporting Information, Material 7 TSS and direction of replication. The asterisk marks the GLI consensus sequence in PTCH necessary for mediating HH/GLI signaling 1. No STAT3 binding regions were found. (*d*) Luciferase reporter assays in combination with site‐directed mutagenesis of predicted GLI and STAT3 binding sites in the PLAT promoter region. Statistical analysis, Student's *t* test; **p < 0.01; *p < 0.05; (*e*) ChIP analysis of EDN2, NRP1 and PLAT for histone H3 acetylated at lysine 27 (H3K27ac) and rabbit IgG (rIgG) in human HaCaT keratinocytes. rIgG served as negative control. IP, immunoprecipitation; (*f*) Human HaCaT keratinocytes were stimulated with Dox for GLI1 induction and IL6. The heatmap represents the methylation level in percent of two GLI1 binding sites (at position −8543 and −8514 relative to the TSS) and one STAT3 binding region (at position −8720 relative to the TSS) as determined by bisulfite sequencing. In each binding site or region three adjacent CpG sites were analyzed. GLI binding sites were biotin‐tagged with a universal sequence as described in 2.
**Figure S5** *In vivo* relevance of Hh‐Il6 cooperation. (*a*) Il6ra and tStat3 expression in BCC‐like lesions of *PtchΔep* mice (upper row) (for Il6ra also, see Fig. 4*c*), *K5creERT;R26SmoM2* mice with a constitutively active SmoM2 mutant (middle row) and *K5cre;Cleg2* mice with skin tumor lesions induced by conditional dominant active GLI2 expression (lower row) 3. Arrow marks nuclear Stat3. scale bars, 25 μm; t, total; (*b*) Western blot analysis (upper panel) and qPCR analysis (lower panel) of Stat3 expression in shStat3‐transduced murine Ptch‐deficient BCC cells (ASZ001) at the start and termination of the allograft. For Western blot analysis protein samples were pooled. β‐Actin served as loading control. Fine black lines indicate cropping of intermediate lanes from the same Western blots. Shcont, scrambled non‐target control shRNA; (*c*) Experimental timeline for Tamoxifen (TAM)‐induced genetic deletion of *Ptch* only or in combination with *Il6ra*. P, postnatal day; (*d)* Quantification of Il6ra‐KO validation by IHC in dorsal skin of control, PtchΔep;Il6ra+/+ and PtchΔep;Il6raΔep mice. Statistical analysis: Student's *t* test; ***p < 0.001; **p < 0.01; ns, not significant.

**Table S1** Chemicals and reagents used for cell treatments
**Table S2** Primer sequences used for qPCR and ChIP‐qPCR analysis
**Table S3** Antibodies used for Western blot, IHC and ChIP analysis
**Table S4** Primer sequences for bisulfite pyrosequencing
**Table S5** Screen of activation of established downstream effectors by secreted factors in HaCaT cells.
