# Supplementary material for: Synergistic cross‐talk of hedgehog and interleukin‐6 signaling drives growth of basal cell carcinoma
Source: Int J Cancer. 2018 Oct 1;143(11):2943–54. doi: 10.1002/ijc.31724 (PMC6282712; doi:10.1002/ijc.31724)
Supplement: Supplementary file 1 — Figure S1 Functionality of shRNA constructs and inhibitors. (a) Knockdown efficiency of shRNA against IL6R (shIL6R#1, shIL6R#2), JAK2 (shJAK2#1, shJAK2#2) and STAT3 (shSTAT3#1, shSTAT3#2) validated by qPCR analysis of mRNA levels (left panels) and by Western blot analysis (right panels) of HaCaT keratinocytes. qPCR signals are given relative to non‐target shRNA (shcont). (b) Western blot analysis of pSTAT3 expression in HaCaT keratinocytes treated with IL6 plus/minus panJAK‐Inh I (1 μM), lestaurtinib (300 nM, 500 nM and 1 μM) and Bayer‐18 (300 nM, 500 nM and 1 μM). (c) Western blot analysis of pSTAT3 levels upon stimulation with the STAT3 activating cytokine Oncostatin M (50 ng/ml) and STATTIC (0.1, 1, 10 and 100 μM). shcont, scrambled non‐target control shRNA; p, phospho; t, total; β‐Actin, β‐Tubulin and tERK served as loading controls. Figure S2 Perturbation of Hh‐Il6‐mediated transformation in NIH/3 T3 cells. (a) Quantification of in vitro transformation assay using Gli1 expressing mouse NIH/3 T3 cells treated with Il6, panJAK‐Inh I (1 μM) or lestaurtinib (300 nM and 500 nM) as indicated. Empty vector not expressing Gli1 served as control. (b) Quantitative analysis of in vitro transformation assay of SAG‐responsive NIH/3 T3 cells upon SAG (100 nM), Il6 or panJAK‐Inh I (1 μM) stimulation. Statistical analysis, Student's t test; ***p < 0.001. Figure S3 Lack of evidence for mutual regulation of GLI1 and STAT3. (a) Western blot analysis of human HaCaT keratinocytes upon Dox and IL6 treatment demonstrated no impact of active IL6/STAT3 signaling on GLI1 protein stability. Additional treatment with the proteasome inhibitor MG132 (25 μM) did not change GLI1 protein levels indicating no regulation by the proteasome machinery. Protein samples from untreated, single or combined treated cells were harvested after 18 hours treatment. (b) Dox‐induced GLI1 expression in human HaCaT keratinocytes is unaffected by knockdown of STAT3 (shSTAT3#1) as revealed by Western blot analysi [file IJC-143-2943-s001.pdf]

Sternberg et al. supplementary figures and information

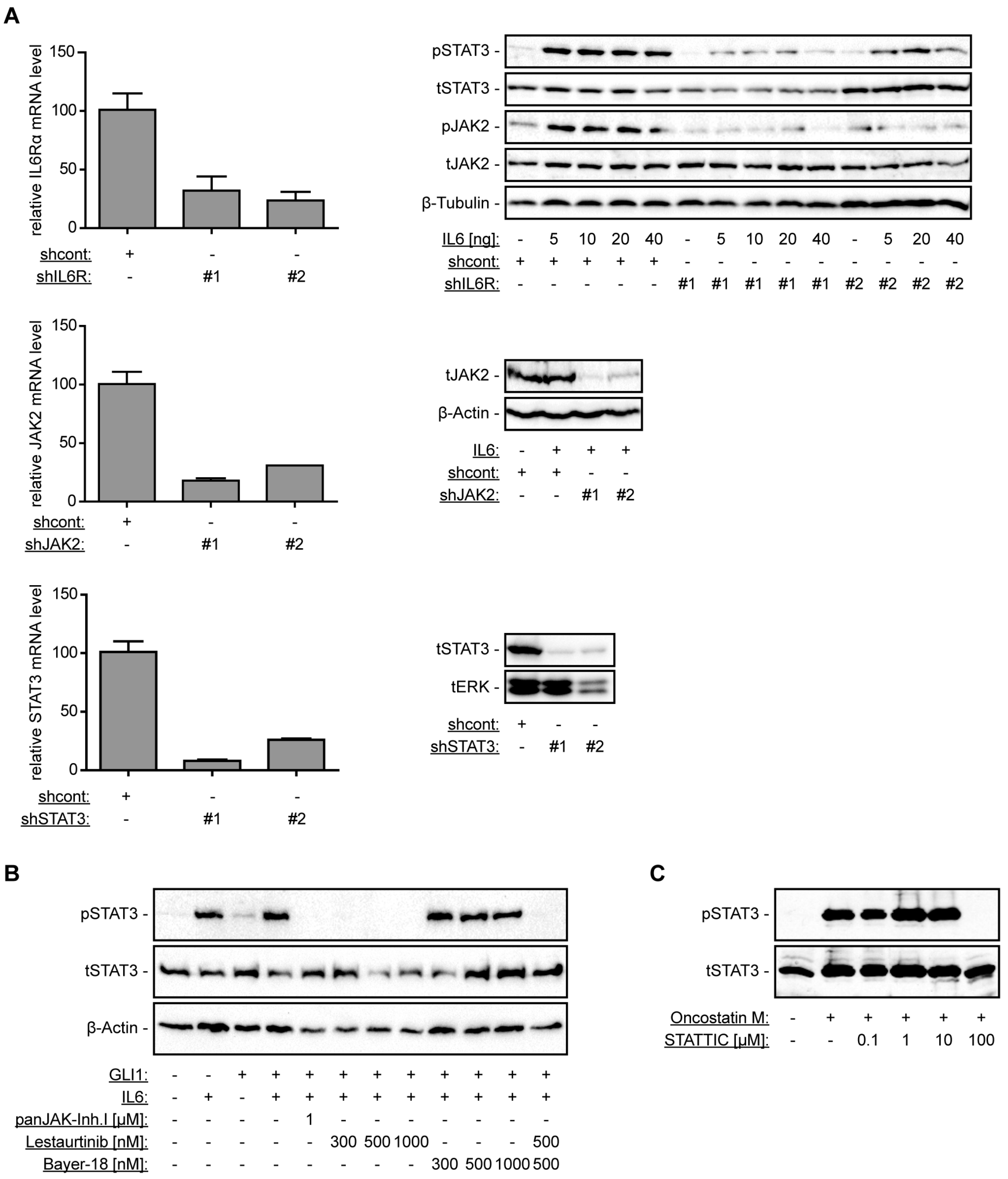

Figure S1: Functionality of shRNA constructs and inhibitors.
A) Knockdown efficiency of shRNA against IL6R (shIL6R#1, shIL6R#2), JAK2 (shJAK2#1, shJAK2#2) and STAT3 (shSTAT3#1, shSTAT3#2) validated by qPCR analysis of mRNA levels (left panels) and by Western blot analysis (right panels) of HaCaT keratinocytes. qPCR signals are given relative to non-target shRNA (shcont).
B) Western blot analysis of pSTAT3 expression in HaCaT keratinocytes treated with IL6 plus/minus panJAK-Inh I (1µM), lestaurtinib (300 nM, 500 nM and 1µM) and Bayer-18 (300

nM, 500 nM and 1μM).
C) Western blot analysis of pSTAT3 levels upon stimulation with the STAT3 activating cytokine Oncostatin M (50 ng/ml) and STATTIC (0.1 μM, 1 μM. 10 μM and 100 μM).
shcont, scrambled non-target control shRNA; p, phospho; t, total; β-Actin, β-Tubulin and tERK served as loading controls.

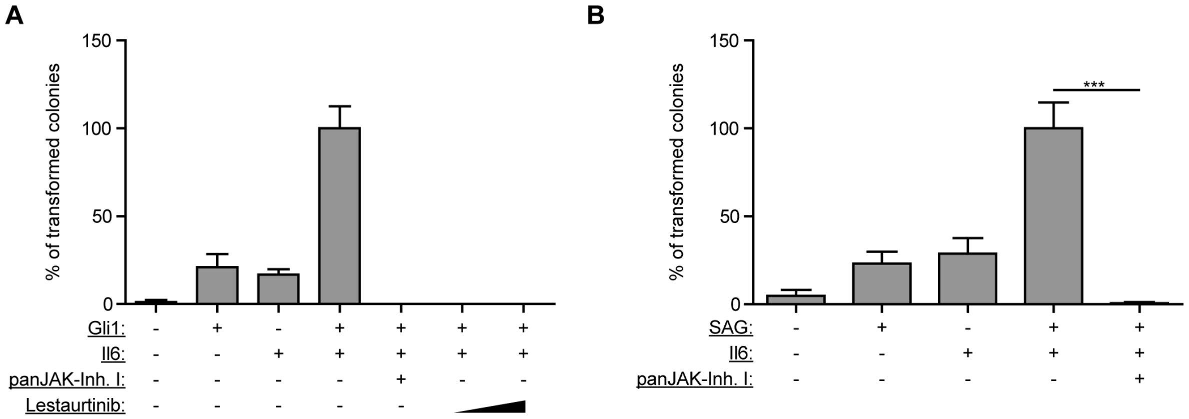

Figure S2: Perturbation of Hh-Il6-mediated transformation in NIH/3T3 cells.
A) Quantification of *in vitro* transformation assay using Gli1 expressing mouse NIH/3T3 cells treated with Il6, panJAK-Inh I (1µM) or lestaurtinib (300 nM and 500 nM) as indicated. Empty vector not expressing Gli1 served as control.
B) Quantitative analysis of *in vitro* transformation assay of SAG-responsive NIH/3T3 cells upon SAG (100 nM), Il6 or panJAK-Inh I (1 µM) stimulation.
Statistical analysis, Student's t-test; ***$p < 0.001$;

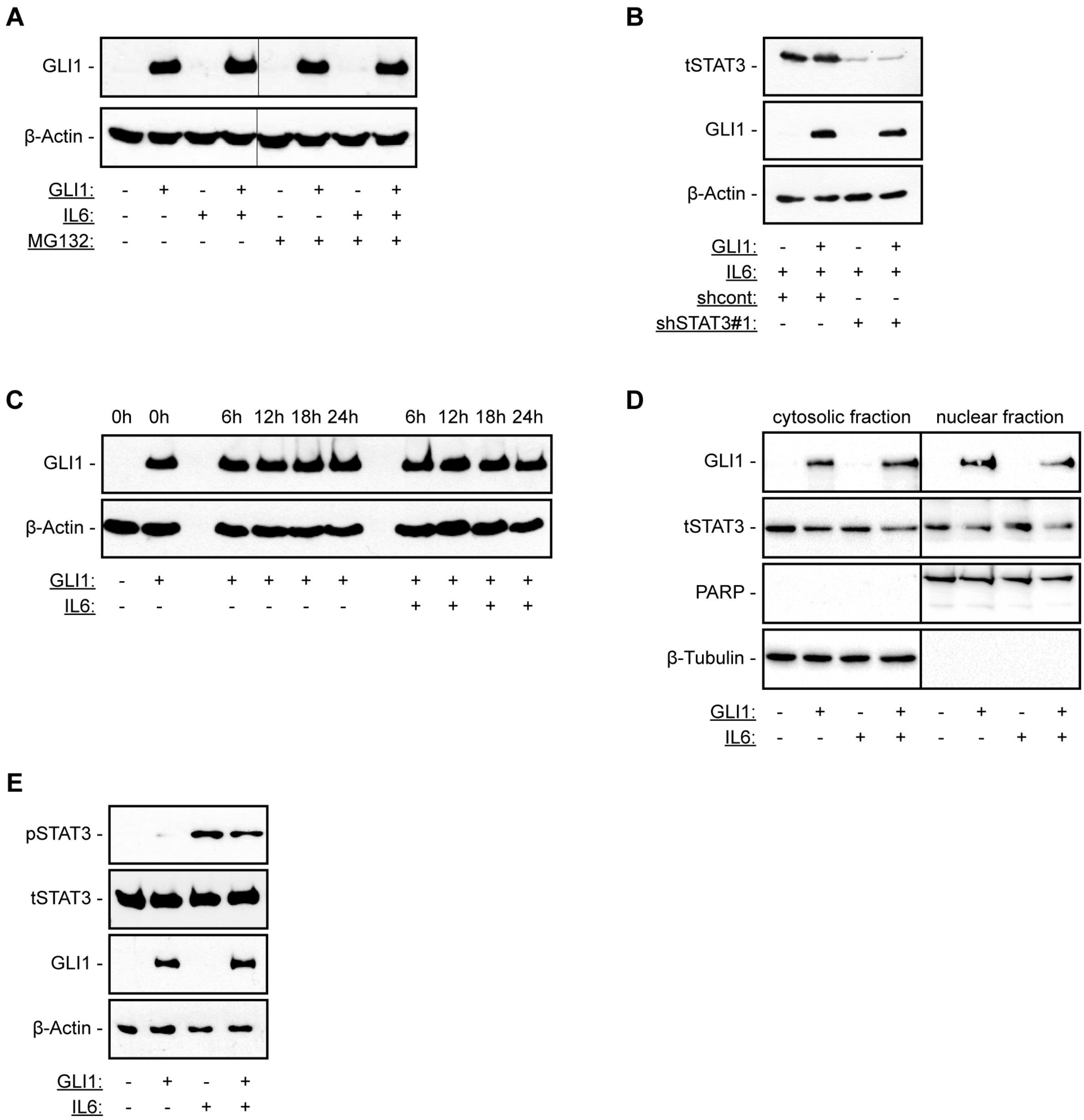

Figure S3: Lack of evidence for mutual regulation of GLI1 and STAT3.
A) Western blot analysis of human HaCaT keratinocytes upon Dox and IL6 treatment demonstrated no impact of active IL6/STAT3 signaling on GLI1 protein stability. Additional treatment with the proteasome inhibitor MG132 (25 µM) did not change GLI1 protein levels indicating no regulation by the proteasome machinery. Protein samples from untreated, single or combined treated cells were harvested after 18 hours treatment.
B) Dox-induced GLI1 expression in human HaCaT keratinocytes is unaffected by knockdown of STAT3 (shSTAT3#1) as revealed by Western blot analysis.
C) Western blot analysis of GLI1 expressing human HaCaT keratinocytes showed no effect of IL6 signaling on GLI1 protein levels and time-resolved expression analysis revealed no changes in GLI1 protein levels over time. Human HaCaT keratinocytes were pre-treated with Dox for 48 hours and the time course was started by adding IL6. GLI levels were detected after 0, 6, 12, 18 and 24 hours. Control cells were included for the 0 hour timepoint.

D) Western blot analysis of cytosolic and nuclear proteins isolated from human HaCaT keratinocytes demonstrated no impact of IL6/STAT3 signaling on nuclear import or export of GLI1 protein. Human HaCaT keratinocytes were pre-treated with Dox for 48 hours and then stimulated with IL6 for 18 hours. β-Tubulin and PARP served as loading controls for cytoplasmic and nuclear proteins, respectively.
E) Human HaCaT keratinocytes with concomitant HH and IL6 signaling showed that GLI1 expression did not change tSTAT3 and pSTAT3 levels. Furthermore, IL6 treatment did not affect tSTAT3 protein levels. Human HaCaT keratinocytes were pre-treated with Dox for 48 hours and then either stimulated by Dox, IL6 or a combination of both for 18 hours.
p, phospho; t, total; shcont, scrambled non-target control shRNA; β-Actin, β-Tubulin and PARP served as loading controls; Fine black lines indicate cropping of intermediate lanes from the same Western blots.

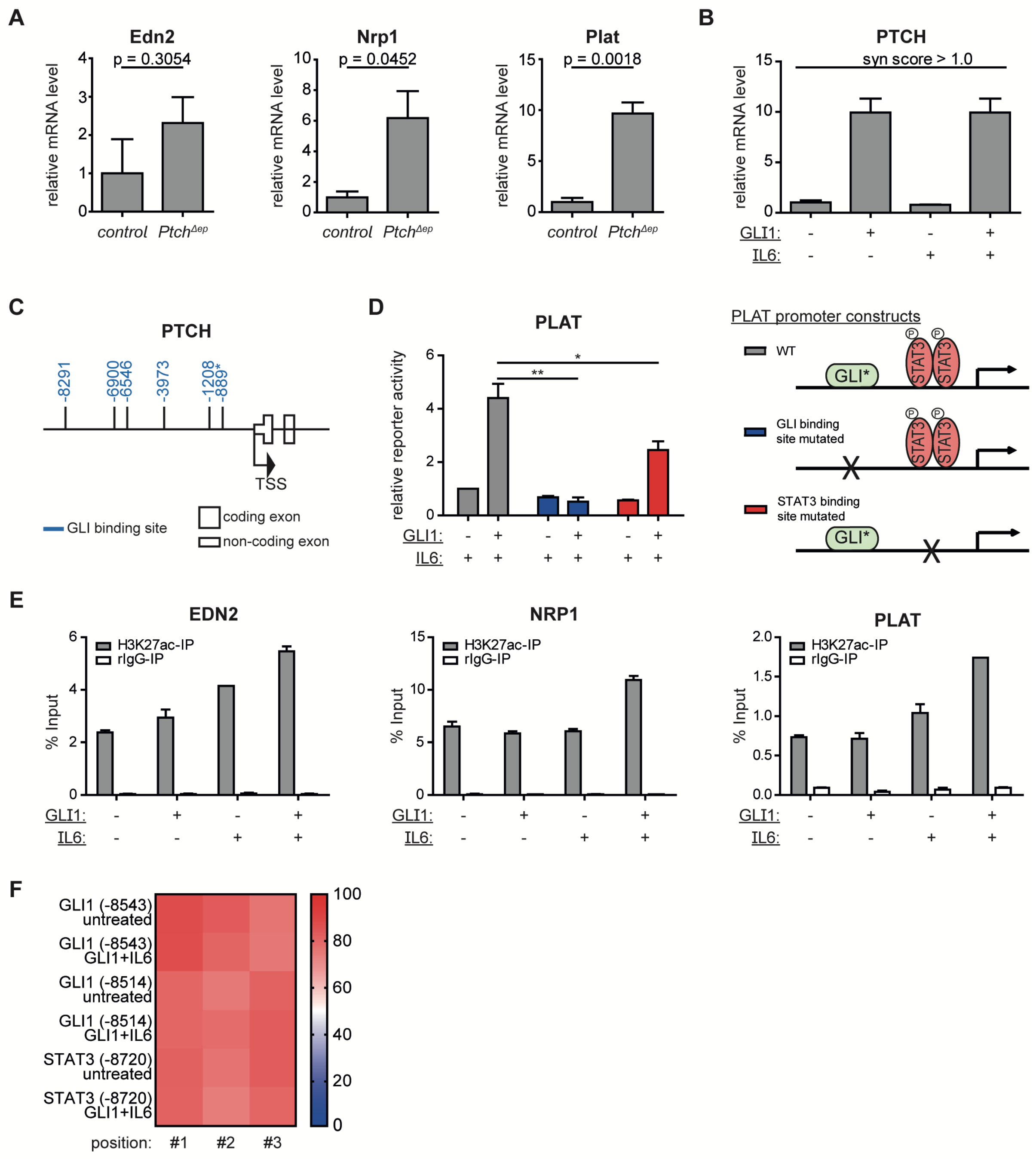

Figure S4: GLI1 and STAT3 interaction at promoters of common HH-IL6 target genes.
A) qPCR analysis of HH-IL6 target gene expression (Edn2, Nrp1 and Plat) in BCC-like lesions derived from mice with epidermal-specific deletion of Ptch (*Ptch*$^{\Delta ep}$) compared to Ptch-proficient control skin (*control*).
B) qPCR analysis of the canonical HH target gene PTCH. Untreated, single (GLI1 or IL6) or simultaneously treated human HaCaT keratinocytes (GLI1+IL6) were analyzed. Synergy score >0.9.
C) *In silico* analysis of the cis-regulatory region of the HH target gene PTCH for the presence of STAT3 binding regions and putative GLI binding sites. Numbers show the relative start positions of GLI binding sites (blue) to the transcription start site (TSS). The arrow shows the

TSS and direction of replication. The asterisk marks the GLI consensus sequence in PTCH necessary for mediating HH/GLI signaling [1]. No STAT3 binding regions were found.
D) Luciferase reporter assays in combination with site-directed mutagenesis of predicted GLI and STAT3 binding sites in the PLAT promoter region. Statistical analysis, Student's t-test; $**p < 0.01$; $*p < 0.05$;
E) ChIP analysis of EDN2, NRP1 and PLAT for histone H3 acetylated at lysine 27 (H3K27ac) and rabbit IgG (rIgG) in human HaCaT keratinocytes. rIgG served as negative control. IP, immunoprecipitation;
F) Human HaCaT keratinocytes were stimulated with Dox for GLI1 induction and IL6. The heatmap represents the methylation level in percent of two GLI1 binding sites (at position -8543 and -8514 relative to the TSS) and one STAT3 binding region (at position -8720 relative to the TSS) as determined by bisulfite sequencing. In each binding site or region three adjacent CpG sites were analyzed. GLI binding sites were biotin-tagged with a universal sequence as described in [2].

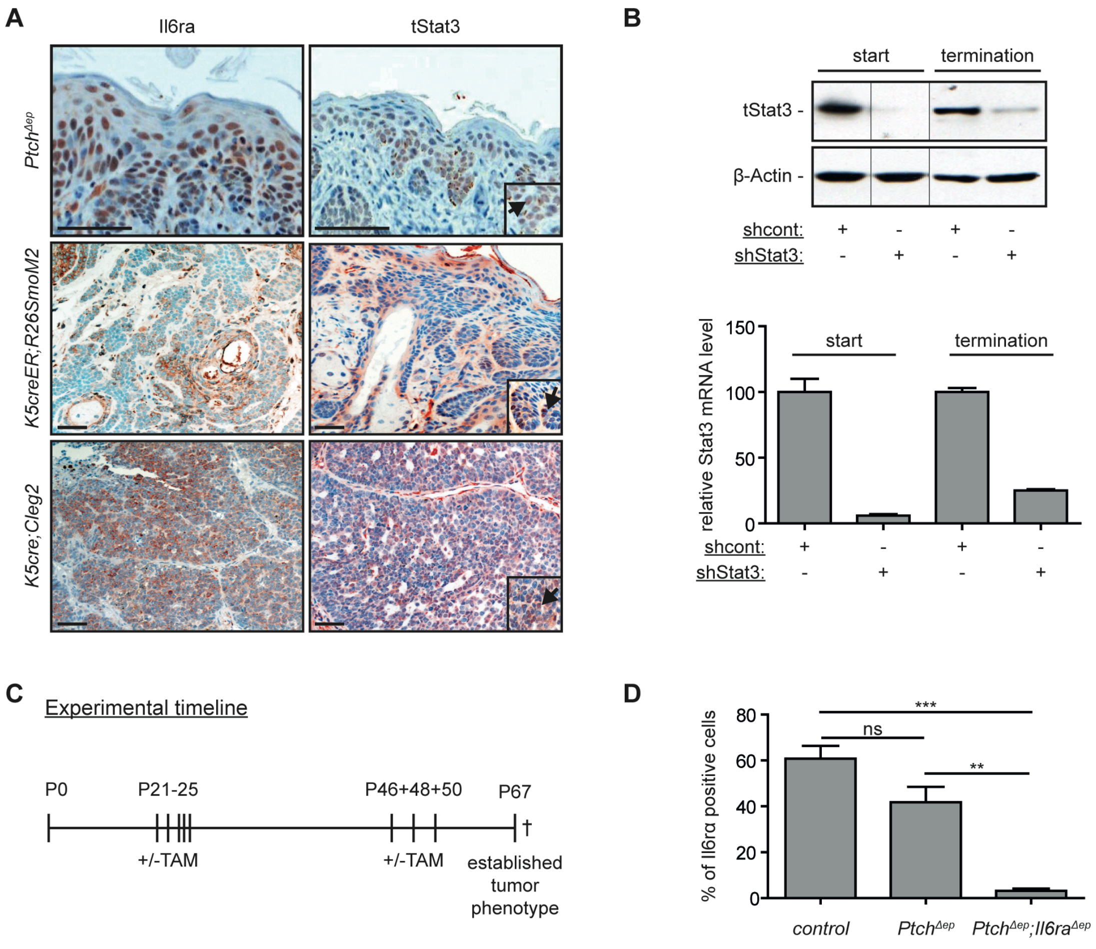

Figure S5: ***In vivo*** **relevance of Hh-Il6 cooperation.**
A) Il6ra and tStat3 expression in BCC-like lesions of *Ptch*$^{\Delta ep}$ mice (upper row) (for Il6ra also see Figure 4C), *K5creER*^{T}*;R26SmoM2* mice with a constitutively active SmoM2 mutant (middle row) and *K5cre;Cleg2* mice with skin tumor lesions induced by conditional dominant active GLI2 expression (lower row) [3]. Arrow marks nuclear Stat3. scale bars, 25 µm; t, total;
B) Western blot analysis (upper panel) and qPCR analysis (lower panel) of Stat3 expression in shStat3-transduced murine Ptch-deficient BCC cells (ASZ001) at the start and termination of the allograft. For Western blot analysis protein samples were pooled. β-Actin served as loading control. Fine black lines indicate cropping of intermediate lanes from the same Western blots. shcont, scrambled non-target control shRNA;
C) Experimental timeline for Tamoxifen (TAM)-induced genetic deletion of *Ptch* only or in combination with *Il6ra*. P, postnatal day;
D) Quantification of Il6ra-KO validation by IHC in dorsal skin of control, Ptch$^{\Delta ep}$;Il6ra$^{+/+}$ and Ptch$^{\Delta ep}$;Il6ra$^{\Delta ep}$ mice. Statistical analysis: Student's t-test; ***$p < 0.001$; **$p < 0.01$; ns, not significant;

Supplementary information

K5cre;Cleg2 **mice:**

K5cre;Cleg2 mice develop BCC-like tumor lesions in response to cre-mediated epidermal expression of a dominant active form of human GLI2 [3, 4]. Dorsal skin specimen were isolated from transgenic, tumor bearing and control mice and stained for Il6ra and Stat3.

K5creER;R26SmoM2 **mice:**

K5creER^T;R26SmoM2 mice were generated as described by Eberl et al. [5]. Tumor lesions were induced by administration of TAM resulting in activation of oncogenic SmoM2 expression in the basal layer of the epidermis and in hair follicles [4-6]. Dorsal skin specimen were isolated from transgenic, tumor bearing and control mice and stained for Il6ra and Stat3.

Extended Material and Methods

Subcellular localization assays

For subcellular localization assays, cells were harvested in cytosolic fraction buffer (10 mM Hepes, 1.5 mM $MgCl_2$, 10 mM KCl, 0.5 mM DTT, 0.2 mM PMSF, 2µM Staurosporin and 50 mM NaF) and incubated on ice for 15 min. Subsequently, 0.1% NP-40 were supplemented followed by incubation for 5 min on ice. Centrifugation enriched the cytosolic proteins in the supernatant. The pellet was dissolved in nuclear fraction buffer (20 mM Hepes, 420 mM NaCl, 1.5 mM $MgCl_2$, 0.2 mM EDTA, 0.5 mM DTT, 0.2 mM PMSF, 20% Glycerol, 2µM staurosporine and 50 mM NaF) and incubated on ice for 20-30 min followed by repeated centrifugation leading to accumulation of nuclear proteins in the supernatant. Buffers were supplemented with protease inhibitor cocktail (Roche, 04693159001) and Phos-STOP phosphatase inhibitor cocktail (Roche, 04906837001). Protein concentrations were determined using a Pierce BCA protein assay (Thermo Fisher Scientific, 23227) according to the manufacturer's instructions.

References

1. Agren M, Kogerman P, Kleman MI, Wessling M, Toftgard R. Expression of the PTCH1 tumor suppressor gene is regulated by alternative promoters and a single functional Gli-binding site. *Gene* 2004;**330**: 101-14.

2. Royo JL, Hidalgo M, Ruiz A. Pyrosequencing protocol using a universal biotinylated primer for mutation detection and SNP genotyping. *Nat Protoc* 2007;**2**: 1734-9.
3. Pasca di Magliano M, Sekine S, Ermilov A, Ferris J, Dlugosz AA, Hebrok M. Hedgehog/Ras interactions regulate early stages of pancreatic cancer. *Genes Dev* 2006;**20**: 3161-73.
4. Ramirez A, Page A, Gandarillas A, Zanet J, Pibre S, Vidal M, Tusell L, Genesca A, Whitaker DA, Melton DW, Jorcano JL. A keratin K5Cre transgenic line appropriate for tissue-specific or generalized Cre-mediated recombination. *Genesis* 2004;**39**: 52-7.
5. Eberl M, Klingler S, Mangelberger D, Loipetzberger A, Damhofer H, Zoidl K, Schnidar H, Hache H, Bauer HC, Solca F, Hauser-Kronberger C, Ermilov AN, Verhaegen ME, Bichakjian CK, Dlugosz AA, Nietfeld W, Sibilia M, Lehrach H, Wierling C, Aberger F. Hedgehog-EGFR cooperation response genes determine the oncogenic phenotype of basal cell carcinoma and tumour-initiating pancreatic cancer cells. *EMBO molecular medicine* 2012;**4**: 218-33.
6. Xie J, Murone M, Luoh SM, Ryan A, Gu Q, Zhang C, Bonifas JM, Lam CW, Hynes M, Goddard A, Rosenthal A, Epstein EH, Jr., de Sauvage FJ. Activating Smoothened mutations in sporadic basal-cell carcinoma. *Nature* 1998;**391**: 90-2.
