# Supplementary material for: Synergistic cross‐talk of hedgehog and interleukin‐6 signaling drives growth of basal cell carcinoma
Source: Int J Cancer. 2018 Oct 1;143(11):2943–54. doi: 10.1002/ijc.31724 (PMC6282712; doi:10.1002/ijc.31724)
Supplement: Supplementary file 2 — Table S1 Chemicals and reagents used for cell treatments Table S2 Primer sequences used for qPCR and ChIP‐qPCR analysis Table S3 Antibodies used for Western blot, IHC and ChIP analysis Table S4 Primer sequences for bisulfite pyrosequencing Table S5 Screen of activation of established downstream effectors by secreted factors in HaCaT cells. [file IJC-143-2943-s002.pdf]

Supplementary tables:

Table S1: Chemicals and reagents used for cell treatments

Reagent / Chemical	Manufacturer	Product number
Bayer-18	Synkinase	SYN-1130
Doxycycline (Dox)	Sigma	D9891
Epidermal Growth Factor (EGF)	Sigma	E9644
Interferon α (IFNα)	Immunotools	11343504
Interferon γ (IFNγ)	Immunotools	11343534
Interleukin-11 (IL11)	Peprotech	200-11
Interleukin-17A (IL17A)	Peprotech	200-17
lestaurtinib	Tinib Tools	L100
MG132	Sigma	C2211
Oncostatin M (OSM)	Immunotools	11344223
panJAK Inhibitor I (panJAK-Inh I)	Calbiochem	420099
Interleukin-6 (IL6; human)	Peprotech	200-06
Interleukin-6 (Il6; mouse)	Peprotech	216-16
Smoothened agonist (SAG)	Axxora	BV-1939
STATTIC	Sigma	S7947
Transforming growth factor β3 (TGFβ3)	Peprotech	100-36E
Tumor necrosis factor α (TNFα)	Immunotools	11343013

Table S2: Primer sequences used for qPCR and ChIP-qPCR analysis

**Human Gene**	**Forward primer (5´-3´)**	**Reverse primer (5´-3´)**	**Application**
RPLP0	(Eberl et al, 2012)		qPCR
EDN2	CCACTTGGACATCATCTG GGTGAACA	TGGAAATGTCCCTCAG CCTTTGGAG	qPCR
IL6R	CCCCCACTCCTGGAACTC ATCTTTC	ACTCGCCTTGCCCGAA CTCCTC	qPCR
JAK2	TGGAGATGTGCCGGTATG ACCCTCT	TACGCCGACCAGCACT GTAGCACAC	qPCR
NRP1	TCAGGGAAACACCAACC CCACAGA	CACCATACCCAACATT CCAGAGCAAGG	qPCR
PTCH	(Eberl et al, 2012)		qPCR
PLAT	GGAAATCCATGCCCGATT CAGAAGA	CTCGCTGCAACTTTTG ACAGGCACT	qPCR
STAT3	AGGCGTCACTTTCACTTG GGTGGAG	TCCTGGCTCTCTGGCC GACAATACT	qPCR
EDN2	GGGAGGGGAAAGCAGGG ATGAAC	TCAGCCAGGGCAGGA ACAGAGG	ChIP-qPCR
NRP1	CCCTGATTTCCACACCCC TCCCTA	CCGAGATTTGGTGCCC GTTCC	ChIP-qPCR
PLAT	TGGGGAAAACGGGAACA ATAGTCTGG	TGCATCCTTGGCTCTG GGAAACA	ChIP-qPCR
PTCH	(Winklmayr et al, 2010)		ChIP-qPCR
**Mouse Gene**	**Forward primer (5´-3´)**	**Reverse primer (5´-3´)**	**Application**
Rplp0	(Eberl et al, 2012)		qPCR
Stat3	TGTAGAGCCATACACCAA GCAGCAGC	GGTCTTCAGGTACGGG GCAGCAC	qPCR
Edn2	TCTGCCTGTGCTACCTTCT GCCATC	CAGGGATGGCCTTTCT TGTCACCTC	qPCR
Nrp1	TGATGAAACAGGGAGCA CTCCAGGATA	ACAACTCCACAGACTG CACCCAGGA	qPCR
Plat	CAGTCCAAGCAATGTGCC CAAGAGA	TCAGCCGGTCAGAGA AGAATGGAGA	qPCR

Table S3: Antibodies used for Western blot, IHC and ChIP analysis

Antigen, antibody	Cat.No.	Manufacturer	Dilution	Application
GLI1, V812	2534	Cell Signaling Technology	1:1000	Western blot
GLI1, C-18	sc-6152	Santa Cruz Biotechnology	1:1000	Western blot
STAT3	610189	BD Biosciences	1:5000	Western blot
Phospho-STAT3, Tyr705	9131	Cell Signaling Technology	1:1000	Western blot
JAK2, D2E12	3230	Cell Signaling Technology	1:1000	Western blot
Phospho-JAK2, C80C3	3776	Cell Signaling Technology	1:1000	Western blot
SUFU, C-15	sc-10933	Santa Cruz Biotechnology	1:1000	Western blot
β-Tubulin, 9F3	2128	Cell Signaling Technology	1:1000	Western blot
β-Actin, C4	sc-47778	Santa Cruz Biotechnology	1:3000	Western blot
PARP	9542	Cell Signaling Technology	1:1000	Western blot
ERK1/2	9102	Cell Signaling Technology	1:1000	Western blot
IL6R, C-20	sc-661	Santa Cruz	1:200	IHC
Total-STAT3, H-190	sc-7179	Santa Cruz	1:200	IHC
Ki67	NCL-ki67p	Novocastra	1:100	IHC
MYC-Tag, 9B11	2276	Cell Signaling Technology	1:100	ChIP
H3K27ac, D5E4	8173	Cell Signaling Technology	1:100	ChIP
STAT3, C-20	sc-482X	Santa Cruz Biotechnology	5 µg/10 µg chromatin	ChIP
normal mouse IgG	5415	Cell Signaling Technology	used at the same concentration as the test antibody	ChIP
normal rabbit IgG	2729	Cell Signaling Technology		ChIP
normal rabbit IgG	sc-2027X	Santa Cruz Biotechnology		ChIP
Phospho-ERK1/2, E10	9106	Cell Signaling Technology	1:1000	Western blot
Phospho-AKT, D9E	4060	Cell Signaling Technology	1:1000	Western blot
AKT	9272	Cell Signaling Technology	1:1000	Western blot
Phospho-c-JUN, D47G9	3270	Cell Signaling Technology	1:1000	Western blot
JUN	610327	BD Biosciences	1:5000	Western blot

Table S4: Primer sequences for bisulfite pyrosequencing

Target region in EDN2 relative to TSS	Forward primer (5´-3´)	Reverse primer (5´-3´)	Sequencing primer (5´-3´)	Annealing temp.
STAT3 binding region (-8720)	[Btn]GGTTTGAGTTTAGGGGTAGTAGGAGAA	TTACTCACCTCACCACCCAACACA	CACCACCCAACACAC	69.4°C
GLI1 binding site (-8543)	AGGGGTAGTAGGAGAAGTTT	GACGGGACACCGCTGATCGTTTAATCACCTCACCACCCAACACA	AGTTTGGTTTTAGTTTTTAGG	61.0°C
GLI1 binding site (-8514)	GACGGGACACCGCTGATCGTTTAAGTTTGAGTTTAGGGGTAGTAGGA	CTCCCACCCTAAATCATTACT	CTCACCACCCAACAC	63.4°C

TSS, transcription start site; Btn, Biotin tag;

Table S5: Screen of activation of established downstream effectors by secreted factors in HaCaT cells

Secreted factors	Downstream effectors							
	pSTAT1	pSTAT3	pSTAT4	pJUN	pNFkB	pERK1/2	pJAK2	pAKT
CCL5	n.d.	-	-	-	-	-	-	n.d.
GM-CSF	n.d.	-	n.d.	n.d.	n.d.	n.d.	-	n.d.
IFNα	+	++	n.d.	n.d.	n.d.	n.d.	n.d.	n.d.
IFNγ	++	+	n.d.	n.d.	n.d.	+	n.d.	+
IL6	-	+++	n.d.	n.d.	n.d.	-	n.d.	n.d.
IL8	n.d.	-	n.d.	n.d.	n.d.	-	n.d.	-
IL11	n.d.	-	n.d.	n.d.	n.d.	-	n.d.	n.d.
IL12	n.d.	-	-	-	-	-	-	n.d.
IL13	n.d.	-	n.d.	n.d.	n.d.	-	n.d.	n.d.
IL17	n.d.	-	-	-	+	-	-	+
OSM	n.d.	+++	n.d.	n.d.	n.d.	n.d.	+	n.d.
TGFβ	n.d.	n.d.	n.d.	n.d.	n.d.	+	n.d.	n.d.
TNFα	n.d.	-	-	++	++	++	-	+++

- not detected, + weak phosphorylation, ++ medium phosphorylation, +++ strong phosphorylation, n.d. not determined; cytokines and growth factors highlighted in light blue selected for oncogenic transformation assays;
